# Digital health interventions for psychological outcomes among adolescents and young adults with cancer: a systematic review and meta-analysis

**DOI:** 10.1038/s41746-026-02719-x

**Published:** 2026-05-09

**Authors:** Panpan Xiao, Yihui Wei, Yan Kate Chow, Keary Rui Zhou, Winnie Wan-Yee Tso, Kevin Yi-Lwern Yap, Herbert Ho-Fung Loong, Chi Kong Li, Yin Ting Cheung

**Affiliations:** 1 School of Pharmacy, Faculty of Medicine, The Chinese University of Hong Kong, Hong Kong SAR, China; 2 Department of Paediatrics & Adolescent Medicine, The University of Hong Kong, Hong Kong SAR, China; 3 Department of Paediatrics & Adolescent Medicine, Hong Kong Children’s Hospital, Hong Kong SAR, China; 4 Alice Lee Centre for Nursing Studies, Yong Loo Lin School of Medicine, National University of Singapore, Singapore, Singapore; 5 School of Psychology and Public Health, La Trobe University, Melbourne, Australia; 6 Department of Clinical Oncology, Faculty of Medicine, The Chinese University of Hong Kong, Hong Kong SAR, China; 7 State Key Laboratory of Translational Oncology, The Chinese University of Hong Kong, Hong Kong SAR, China; 8 Department of Paediatrics, Faculty of Medicine, The Chinese University of Hong Kong, Hong Kong SAR, China; 9 Hong Kong Hub of Obstetric and Paediatric Excellence, The Chinese University of Hong Kong, Hong Kong SAR, China

**Keywords:** Diseases, Health care, Medical research, Psychology, Psychology

## Abstract

Adolescents and young adults (AYAs) with cancer show a strong interest in digital health interventions (DHIs); however, limited pooled evidence from randomized controlled trials (RCTs) exists regarding their quantitative benefits in this population. This systematic review and meta-analysis (PROSPERO: CRD42024593656) screened a total of 9949 records from six databases, registers and other sources up to January 5, 2025, and included 31 articles from 25 studies examining DHIs targeting psychological outcomes among AYAs. Most DHIs were conducted via websites (*n* = 13), mobile applications (*n* = 5), wearable devices (*n* = 6), or videoconferencing/telephone (*n* = 6), and interventions were multicomponent and promoted self-management. Meta-analysis showed a significant effect of DHIs on quality of life, anxiety, psychological distress, and social support. Meta-regression revealed that the mode of delivery (instructor-based vs. self-guided) moderated the effects of DHIs on anxiety and depressive symptoms, with instructor-based DHIs producing significantly stronger effects. Future RCTs evaluating the impact of DHIs on cognitive impairment and cost-effectiveness are needed.

## Introduction

Adolescents and young adults (AYAs), defined as individuals aged 15–39 years old^1^, are in a critical stage of cognitive development: the transition from adolescence to emerging adulthood and ultimately to young adulthood^2^. Those diagnosed with cancer during this period face challenges related to the cancer, its treatments, and their late effects. These challenges hinder AYAs’ ability to achieve developmental tasks specific to their age group, such as establishing autonomy, pursuing education, developing romantic relationships, starting a career, and raising children^3,4^. Consequently, their psychological well-being may be significantly threatened, affecting emotional health, social functioning, health behaviors, and cognition^5^. Two recent systematic reviews and meta-analyses reported that the pooled incidences of psychological distress, anxiety, depression, and cognitive impairment (excluding central nervous system tumors) among AYA survivors were 32%, 29%, 24%, 25.75%, respectively^6,7^. Therefore, beyond prioritizing survival outcomes, addressing the unique psychological needs of AYAs and improving their psychological health outcomes are integral to the provision of comprehensive care throughout their cancer care continuum.

Within the field of AYA oncology, programs targeted at psychosocial support^8–10^ and physical activity^11–15^ are the most extensively researched supportive care interventions during and after cancer treatment. Evidence indicates that these approaches can effectively improve health-related outcomes, including anxiety and depressive symptoms, fatigue, physical activity levels, and quality of life (QoL). However, for AYAs, in-person care poses logistical challenges related to time constraints, financial costs, and geographic accessibility^16^. Additionally, stigma and embarrassment associated with openly discussing sensitive topics, such as sexual dysfunction, often deter young people from seeking face-to-face support, leading many to turn to online platforms for information and guidance^17^. Furthermore, time limitation and staffing shortages remain significant challenges to the advancement of AYA cancer survivorship research^18^.

Digital health interventions (DHIs) refer to the use of digital technologies in medicine and other health professions to manage illnesses and health risks and promote wellness. DHIs encompass categories such as mobile health, health information technology, wearable devices, telehealth, telemedicine, and personalized medicine^19^. DHIs have evolved rapidly in recent years because of their accessibility, cost-effectiveness, and flexibility. Moreover, a qualitative study indicated that AYAs preferred cancer survivorship care models that incorporated digital health tools^20^. Reviews published in 2018^21^ and 2020^22^ demonstrated the preliminary feasibility of the applications of DHIs among AYAs with cancer. Three systematic reviews further provide insights regarding the efficacy of DHIs. Zhang et al.^23^ reviewed 28 studies, nine of which focused on AYAs, and found that technology-assisted interventions had a significant overall effect on the emotional health outcomes of childhood and AYA cancer survivors. Chandeying et al.^24^ reviewed 13 studies and demonstrated that online interventions could improve the emotional health outcomes of childhood and AYA cancer survivors (0–39 years of age at diagnosis). Christopherson et al.^25^ reviewed 6 studies and found that active video games could significantly improve QoL and reduce fatigue, but the study designs varied widely and did not permit a pooled analysis of the effect size. Collectively, the age ranges of the participants in these three reviews were broad, and there were no restrictive criteria regarding the defined age of AYAs with cancer. Moreover, several new clinical trials in this field have been published in the last 3 years. Importantly, synthesizing evidence on intervention implementation outcomes (e.g., acceptability, usage, and adherence) is critical for understanding the facilitators and barriers to the application of DHIs among AYAs^26^. Therefore, it is necessary to provide reliable references for the application of DHIs in the AYA field based on current evidence.

To date, no systematic review has comprehensively evaluated DHIs for AYAs with cancer exclusively through randomized controlled trials (RCTs) to draw robust conclusions. Despite the potential benefits of integrating digital health into the cancer care continuum, the limited high-quality evidence from RCTs has likely hindered its widespread adoption. Given the feasibility of DHIs in AYA oncology and emerging evidence from RCTs, an updated synthesis of the current evidence is warranted. This study assesses the efficacy of DHIs on psychological outcomes during and after cancer treatment and evaluates the DHI implementation outcomes, including acceptability and engagement.

## Results

### Study selection

**Fig. 1:**
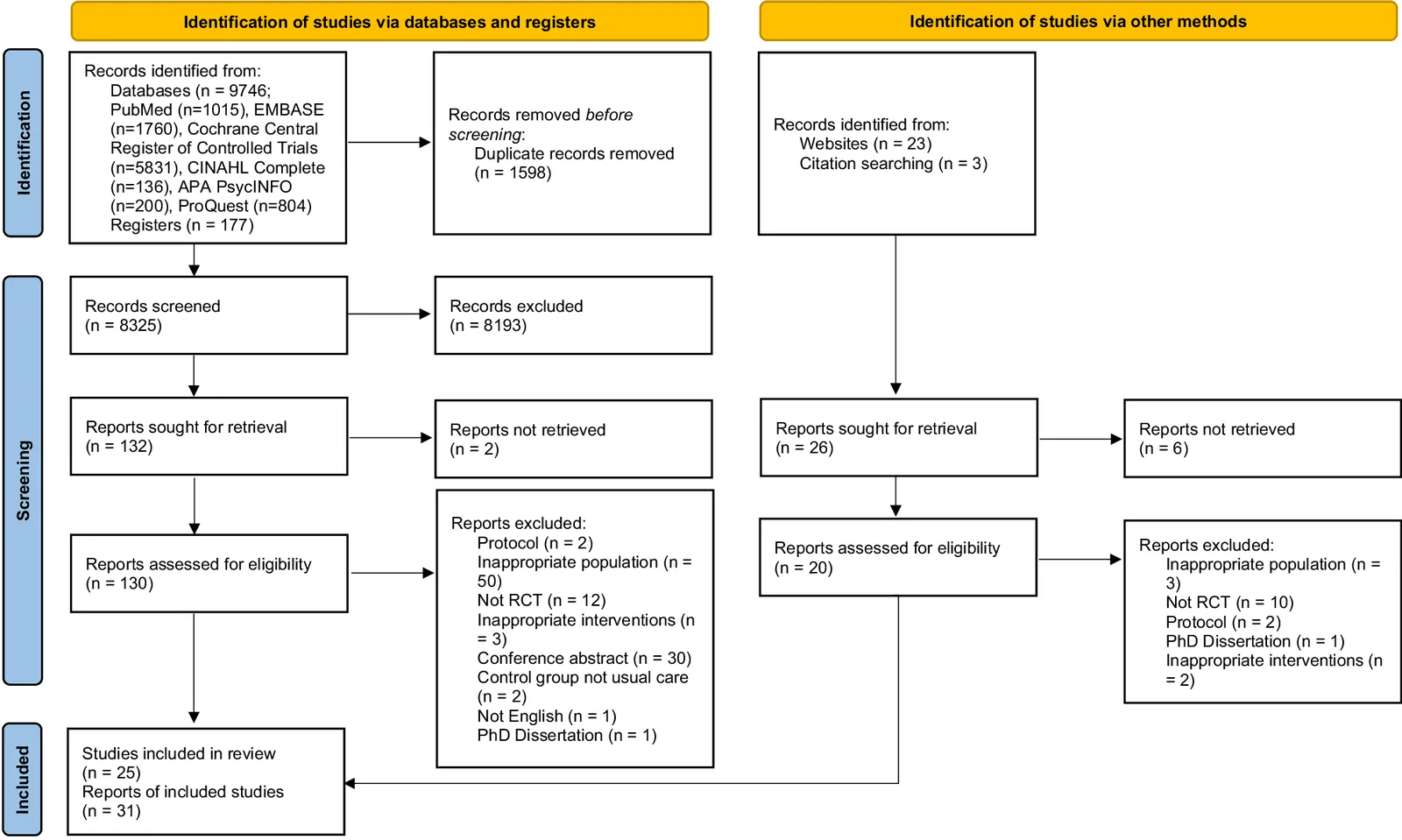
The Preferred Reporting Items for Systematic Reviews and Meta-Analysis 2020 flow diagram of included studies. The PRISMA flow diagram shows the number of studies identified, screened, and selected and the reasons for exclusion.

Our initial research yielded 9949 records. After removing duplicates, a total of 8351 records remained. We excluded 8193 of these records based on the screening of titles and abstracts. Among the remaining 158 articles for full-text review, 31 met the eligibility criteria and were included in the systematic review^27–57^ (Fig. 1). Five studies^32,38,43,53,55^ used the same study population but reported different outcomes.

### Description of included studies

A total of 31 articles representing 25 RCTs were included in the systematic review. The characteristics of the included studies are summarized in Table 1. Twenty-one studies (84%)^28–30,32–34,36–40,44–50,53,55,57^ were conducted in high-income countries, as defined by the 2020 World Bank^58^, and the remaining studies (three from China^35,41,43^ and one from Turkey^51^) were conducted in upper-middle-income countries.

**Table 1:** Characteristics of included studies

Author, year	Country	Sample size	Intervention type	Intervention tools provided	Digital health intervention details	Contact with researcher/health providers	Comparator	Duration	Follow-up	Outcomes and measures
Beale et al. ^27^ & Kato et al.^38^	Netherlands	*N* = 371 *I* = 195 *C* = 176	Website: Re-Mission	Mini-computer provided	Re-Mission (www.re-mission.net): a PC video game that includes destroying cancer cells, managing treatment-related effects	N—100% self-guided	Computer game: Indiana Jones and the Emperor’s Tomb	3 mos	0	Cancer-related knowledge: cancer knowledge scale Cancer-specific self-efficacy: Self-efficacy Scale QoL: PedsQL^TM^ and FACT-G Stress: PSS Sense of control: MHLC
Berg et al. ^28^	USA	*N* = 56 *I* = 38 *C* = 18	1. Smartphone App: Achieving Wellness After Kancer in Early life (AWAKE) app 2. Telephone-based coaching	No	1. AWAKE app: included 8 weekly psychoeducational modules via videos, daily health behavior and mood tracking + 8x homework 2. Weekly coaching calls (8 total) 3. Text messaging twice per week to patients by the coach	Y—Weekly telephone with coach + text messages from coach twice per week	Attention control: 8 weekly educational materials regarding personal finance via text; weekly opportunities for coaching calls; no daily health behavior and mood tracking	2 mos	4 mos	Hope: Adult trait hope scale QoL: RAND-SF-36, FACT-G Depressive symptoms: PHQ-9
Bergstrom et al.^29^	Sweden	*N* = 138 *I* = 72 *C* = 66	Website: Fex-Can Sex program	No	1. Six consecutive psychoeducational modules (12 weeks), including educational and behavior change content + recorded video vignettes + exercises aiming to increase body awareness and acceptance and improve sexual function 2. Online moderated discussion forum	Y—Mostly self-guided, but could discuss with research staff in the forum	Usual care	3 mos	3 mos	Sexual function: PROMIS SexFS version 2.0 QoL: EORTC-QLQ-C30 Body image: Body Image Scale Anxiety and depressive symptoms: HADS Sexual self-efficacy: self-designed Questionnaire
Breaky et al.^30^	Canada	*N* = 81 *I* = 39 *C* = 42	Website: self-management program + monthly calls from health coaches	No	Web-based self-management program: consisting of 12 modules (cancer-specific education, self-management training, transitional skills, and social support strategies) + 4× homework + monthly telephone contact with a trained coach (30–45 min/time)	Y—3x calls from trained coach	Usual care + links to 12 general cancer websites + monthly telephone contact (5 min/time)	3 mos	0	Cancer knowledge: adolescent cancer knowledge questionnaire Self-efficacy: generalized self-efficacy-sherer scale Fatigue: PedsQL Multidimensional Fatigue Anxiety and depressive symptoms: HADS Social support: Perceived social support from friends scale QoL: PedsQL Cancer module
Ehrbar et al.^31,32^	Switzerland	*N* = 51 *I* = 24 *C* = 27	Website: online decision aid (DA)	No	www.fertionco.ch website: information on cancer treatment and its impact on fertility and FP procedures, and an interactive decision-making part with a value clarification exercise about FP options	Y—Received counseling regarding fertility preservation with an oncologist before receiving a link to online DA	Usual care + Counseling only	1 mos	12 mos	Decisional conflict: Decisional conflict scale Decisional regret: Decisional regret scale
Greer et al.^33^	USA	*N* = 45 *I* = 25 *C* = 20	Chatbot: *Vivibot*	No	*Vivibot*: a cognitive and behavioral intervention that focuses on the teaching and practicing of 8 positive psychological skills via texts or videos, including 7 conversational teaching lessons and 7 practice lessons; 6 daily emotion ratings; periodic check-ins on satisfaction with interactions with the chatbot	N—100% self-guided	Waitlist: daily mood ratings only	1 mos	1 mos	Anxiety: PROMIS-anxiety depressive symptoms: PROMIS-depression
Huyghe et al.^34^	France	*N* = 20 *I* = 10 *C* = 10	Website: Banking on Fatherhood (BOF)	No	BOF: presenting medical updates on cancer-related infertility and sperm banking; a knowledge test (the Gamete Game); teaching communication skills via video vignettes	N—100% self-guided	Waitlist	NR	0	Decisional conflict: Decisional conflict scale Knowledge about cancer-related infertility: True/false knowledge test
Jiang et al.^35^	China	*N* = 115 *I* = 60 *C* = 55	1. Mobile App: AI-TA 2. Web-based follow-up	No	1. *AI-TA* mobile app: information covering psychological counseling, coping effectiveness, symptom management, social security, etc.; recorded salon lectures held by breast cancer clinical experts; web-based discussion forums 2. Fortnightly web-based follow-up via private message or calls from healthcare professionals	Y—Fortnightly messages or calls; questions answered in the session of salon lectures Participants were free to get in touch with the healthcare professionals during the 3-month intervention	Access to general information on the AI-TA app + No follow-up conversations	3 mos	0	Psychological distress: Memorial symptom assessment scale-short form self-efficacy: CBI-B Social support: SSRS QoL: FACT-B
Johnson et al.^36^	USA	*N* = 49 *I* = 26 *C* = 23	Fitness tracker: Fitbit Flex + Fitbit app + Facebook group + PA “buddy”	Activity tracker provided	1. A wrist-worn PA tracking device (Fitbit) + Fitbit app 2. Facebook group: moderated by research staff, discuss experiences related to PA goals and Fitbit use, post once a day (4 total in a week) from research staff 3. Individualized goal setting 4. PA “buddy”: provide encouragement and support to patients 5. Text messages every other day: meet basic psychological needs	Y—Weekly brief (≤5 min) goal-setting communication with research staff via text (primarily) or phone + text message every other day during the intervention	Fitbit and Fitbit app only (evidence have shown not having an impact on physical activity)	3 mos	0	PA: ActiGraph GT3X+ QoL: PROMIS Physical Function Fatigue: The Fatigue Symptom Inventory Sleep: Pittsburgh Insomnia Symptom Questionnaire
Jones et al.^37^	USA	*N* = 65 *I* = 35 *C* = 30	Multimedia CD-ROM	No	Interactive multimedia CD-ROM with text, graphics, multiple videos, music, voiceover, and games + manual (specific information needs of adolescents with solid tumors)	N—100% self-guided	Handbook	3 mos	0	QoL: Pediatric Oncology Quality of Life Scale Self-efficacy: Self-efficacy Scale Cancer Knowledge: Cancer Knowledge Questionnaire Coping styles: KIDCOPE Sense of control: MHLC
Kirchhoff et al.^39^	USA	*N* = 86 *I* = 45 *C* = 41	Website: HIAYA CHAT	No	HIAYA CHAT: health insurance education program delivered by a patient navigator, 4 sessions, 30–40 min each, virtually delivered (Zoom) every other week over 2 months; insurance booklet	Y—4 × 30-40 min video conferences	Usual navigation care	5 mos	0	Stress: Perceived Stress Scale Health Insurance Literacy: Health Insurance Literacy Measure Cancer-related financial hardship: COmprehensive Score for financial Toxicity
Lang et al.^40^	Canada	*N* = 26 *I* = 8 *C* = 18	Website: *cancerchatcanada.ca*, CCC	No	1. Online, synchronous chat groups (OSGs, real-time text-only conversations): provide compelling and relevant discussion content to stimulate greater sharing of unique AYA psychosocial challenges with peers 2. Video: clips from an AYA-created film, *Wrong Way to Hope*, focus on sharing feelings and building connections	Y - research staff provided discussion content to participants	Waitlist	2.5 mos	6.5 mos	Anxiety and depressive symptoms: HADS coping self-efficacy: CBI-B
Li et al.^42,43^	China	*N* = 95 *I* = 47 *C* = 48	Wearable electronic device + WeChat group	Activity tracker provided	1. Exercise method: using the wearable device and supporting app, warm up and stretch for 10–15 min, then perform walking exercise and stretch finally, walk 3× per week (20–30 min/time) during the first 2 weeks, walk 4× per week (30–40 min/time) in week 3 and week 4, walk 5 times per week (30–40 min/time) during the last 4 weeks; complete an exercise diary after each exercise 2. WeChat group: post 1–2 questions for patients to answer and discuss each week to promote interaction	Y—8x video calls (10–15 min) regarding health counseling with participants before intervention to confirm the exercise prescription, and 4× video calls (10–15 min) after intervention to discuss the completion of sports goals	Usual care	2 mos	3 mos	QoL: FACT-G Anxiety and depressive symptoms: HADS Social support: SSRS Self-efficacy: General Self-efficacy Scale Sleep: Pittsburgh Sleep Quality Index PA: IPAQ
Li et al.^41^	China	*N* = 90 *I* = 45 *C* = 45	Online-based peer support	No	Online peer support: peer supporters shared cancer experience during cancer treatment, assisted participants in exploring solutions to their problems through example and practical experiences, and applied empathy skills with participants using digital tools and platforms, once a week (90–120 min/time), 8 in total (7 times were online, 1 time was offline) WeChat group: co-managed by peer supporters and research staff; information support provided by peer supporters once every 2 weeks (4 total)	Y—online chat group moderated by research staff and peer supporters	Usual care	2 mos	3 mos	Psychological distress: Distress Thermometer Anxiety and depressive symptoms: HADS Sense of Peer Support: Cancer Peer Support Scales
Micaux et al.^44^	Sweden	*N* = 124 *I* = 64 *C* = 60	Website: Fex-Can Fertility program	No	1. Six successive psychoeducational modules (12 weeks): cover known aspects of fertility distress (Fertility after cancer, Handling anxiety, Trying to have children after cancer, My own health and my child’s health, Not being able to have biological children, and Relationships) 2. Written and filmed survivor stories, interactive quizzes, and a discussion forum (moderated by research staff and with clinical expertise in nursing and psychology)	Y—Mostly self-guided but discussed with research staff in the forum	Usual care	3 mos	3 mos	QoL: EORTC-QLQ-C30 Anxiety and depressive symptoms: HADS Fertility self-efficacy: self-designed questionnaire Fertility distress: RCAC
Rabin et al.^45^	USA	*N* = 18 *I* = 8 *C* = 10	Website: adapted the PA website based on the Step into Motion website	No	PA website: entered weekly PA goal, logged PA performed, completed the monthly questionnaire; discussed on the online forum	Y—Mostly self-guided + automated feedback reports + weekly emails asking if participants experienced specific physical symptoms; if yes, research staff contacted the participants	Information about three publicly available websites: www.stupidcancer.com, www.planetcancer .org), www.cancercare.org, the websites don’t provide PA information	3 mos	0	PA: Seven-day Physical Activity Recall Mood: Profile of Mood States Fatigue: Fatigue domain of Profile of Mood States
Rabin et al.^46^	USA	*N* = 35 *I* = 19 *C* = 16	Telephone-based PA intervention + activity tracker + CD-based meditation intervention	Activity tracker provided	1. Telephone-based PA intervention: weekly call (mean = 19.0 min) by project director with participants (12 total) for behavioral coaching 2. Mindfulness CD: containing sitting meditation, body scan, and yoga stretches, practice 4 days a week 3. Monthly call for 3 months to assist participants with maintaining PA and meditation 4. Online forum moderated by the research staff to allow connection among participants	Y—12× weekly calls from research staff for PA intervention + 3× monthly booster calls from research staff	Waitlist	3 mos	3.5 mos	PA: Seven-day Physical Activity Recall & Accelerometer Mood: Profile of Mood States
Sansom-Daly et al.^47^	Australia	*N* = 30 *I* = 19 *C* = 11	Videoconference: Recapture Life intervention	No	Online Recapture Life intervention: six, once-weekly, 90 min small-group (3–5 AYAs per group) sessions, facilitated by a psychologist; group explored common cancer survivorship experiences and relevant evidence-based CBT coping strategies every week (6 total); participants received the Recapture Life workbook containing psycho-education content reflective of the online session + home-practice activities to practice coping strategies	Y—6x discussions with psychologists on the videoconferencing platforms	Waitlist	1.5 mos	9 mos	QoL: Impact of Cancer Scale Depressive and anxiety symptoms: Depression, Anxiety and Stress Scale-Short Form Coping strategies: KIDCOPE Family functioning: McMaster Family Assessment Device
Shin-Cho et al.^48^	USA	*N* = 25 *I* = 15 *C* = 10	Online expressive writing	No	A website link to read positive messages (A compilation of three quotes, Poem“What Cancer Cannot Do”, Short Story-Responding to Adversity) in the first 3 weeks; expressively write about stress and coping, emotional disclosure, and benefit finding in the last 3 weeks (30 min each)	Y—3× instructions provided by research staff	Read neutral messages (Healthy eating, Physical activity, Getting organized) in the first 3 weeks; write about daily life in the last 3 weeks (30 min each)	1.5 mos	0	QoL: Seven-item FACT Fatigue: PROMIS-Fatigue Depressive symptoms: PROMIS-Depression Anxiety: PROMIS-Anxiety
Snaman et al.^49^	USA	*N* = 50 *I* = 25 *C* = 25	Video: Advance care planning (ACP) video	No	ACP video via a tablet or Zoom: introduce the concept of ACP and 3-goal framework (life-prolonging care, selective care, and comfort care) + visual images	N—100% self-guided	Usual care: verbal description of the three types of care	NR	3 mos	Decisional conflict: 4-item SURE (Sure of myself; Understand information; Risk-benefit ratio; Encouragement) screening test
Tock et al.^50^	Canada	*N* = 26 *I* = 13 *C* = 13	Videoconference + Fitbit app + activity tracker + discussion with kinesiologist	Activity tracker provided	*Lymfit* intervention (via videoconference) 1. First appointment: study package consisting of a Fitbit, exercise resistance bands, and mailed to participants 2. Second appointment: set up the Fitbit in the smartphone and a private group “Lymfit lounge” in the Fitbit app for connection 3. Third appointment: discussion exercise prescription, expectations, and any concerns 4. Bi-weekly follow-up for 12 weeks (5 meetings in total with a kinesiologist via videoconferencing to discuss progress/modification of exercise prescription as needed)	Y—8× discussions with kinesiologists via videoconference	Waitlist: usual care	3 mos	0	PA: Godin-Shephard leisure-time physical activity questionnaire (LTPA-Q) Anxiety: PROMIS-Anxiety Depressive symptoms: PROMIS-Depression Fatigue: PROMIS-Fatigue Sleep disturbance: PROMIS-Sleep disturbance Social integration: PROMIS-Ability to participate in social roles and activities QoL: PROMIS-Total
Uluhan et al.^51^	Turkey	*N* = 46 *I* = 23 *C* = 23	Website: Re-Mission	Mini-computer provided	Re-Mission (www.re-mission.net): a PC video game that includes destroying cancer cells, managing treatment-related effects	N—100% self-guided	Usual care	3 mos	0	Fatigue: Scale for the assessment of fatigue in pediatric oncology patients aged 13-18 QoL: FACT-G
Valle et al.^55,56^ & 2017	USA	*N* = 86 *I* = 45 *C* = 41	Facebook-based intervention (FITNET): behavioral lessons via Facebook + Activity tracker + mobile app + weekly message + website + PA diary + Facebook group discussion	Activity tracker provided	FITNET: received pedometer to record total daily steps and introductory email about the intervention goal and recommendation; joined the Facebook group, study administrator posted discussion questions (twice a week in the first 4 weeks, once a week in the last 8 weeks), links to videos, related articles, electronic PA resources; weekly messages about behavioral lessons with more specific guidance on PA and behavioral strategies; access to a separate study website sending a weekly reminder to set an exercise goal and log daily PA	Y—Introductory email about the intervention goal and recommendation; weekly automated feedback about exercise progress; research staff posted 16× discussion questions; research staff answered questions and offered encouragement and support every week; weekly email reminder	Facebook-based self-help comparison: received a pedometer to record total daily steps and an introductory email about the intervention goal and recommendation; joined the Facebook group; weekly messages about basic information and tips related to PA; several links to publicly available websites	3 mos	0	PA: Godin Leisure Time Exercise Questionnaire QoL: FACT-G Self-efficacy for PA: Self-Efficacy and Exercise Habits Survey Social support for exercise: Social Support and Exercise Survey
Valle et al, 2023a & 2023b & 2024	USA	*N* = 280 *I* = 140 *C* = 140	Mobile PA intervention: Improving Physical Activity after Cancer Treatment (IMPACT)	Activity tracker provided	IMPACT intervention 1. Mobile responsive website: provided weekly lessons about psychoeducation and behavioral skills training, adaptive PA goals, and weekly tailored feedback 2. Text message: received 5× texts each week in the first 24 weeks, 1× text per week after week 24 3. Facebook group for peer support: participants were encouraged to post comments about PA. Interventionists responded to questions and posted prompts up to 5 times per week 4. A Fitbit tracker + smart scale + kickoff individual telephone or videochat session	Y—weekly tailored automated feedback + 5 text messages per week + 1× telephone with research staff about the introduction of the intervention	Self-help intervention: received an activity tracker and companion mobile app, and an email with instructions on setting up Fitbit; received a cellular-enabled smart scale; voluntarily participated in the initial individual telephone or video chat session; joined the Facebook group, but the interaction was not specifically encouraged	3 mos	6 mos	PA: ActiGraph GT3X + & Godin Leisure Time Exercise Questionnaire Sedentary behaviors: Sedentary Behavior Questionnaire QoL: SF-36
Zhang et al.^57^	USA	*N* = 17 *I* = 10 *C* = 7	Telephone or web-based videoconference intervention: Mind Your Total Health (MYTH)	No	MYTH: weekly received one session (8 in total) of technology-assisted cognitive-behavioral therapy (tCBT) via video and text and character-driven video storyline; a brief check-in call (10–15 min) with a trained coach via telephone or secure, videoconference platforms; weekly mood and safety check-ins delivered by study coaches	Y—One brief check-in call from the study coach + weekly mood and safety check-ins from the study coach via telephone or videoconference platforms	Active control 1. BeatingTheBlues (BtB, www.beatingtheblues.co.uk): 8- session core CBT components, including cognitive restructuring, behavioral activation, and problem-solving. BtB did not include any customization to address the unique needs of AYAs and cannot be changed without substantial cost, time and effort 2. Brief check-in calls (10–15 min) after completion of each session	2 mos	0	Depressive symptoms: PHQ-9 Anxiety: GAD-7

*NR* not report, *mos* months, *PC* personal computer, *PedsQLTM* Pediatric Quality of Life InventoryTM, *FACT-G* Functional Assessment of Cancer Therapy- General, *MHLC* Multidimensional Health Locus of Control, *SF-36* 36-Item Short Form Health Survey, *PHQ-9* Patient Health Questionnire-9, *HADS* Hospital Anxiety and Depression Scale, *PROMIS* Patient-Reported Outcomes Measurement Information System, *CBI-B* Cancer Behavior Inventory-Brief Version, *SSRS* Social Support Rating Scale, *KIDCOPE* Kid’s Coping Strategies Checklist, *IPAQ* International Physical Activity Questionnaire, *EORTC-QLQ-C30* European Organization for Research and Treatment of Cancer Quality of Life Questionnaire, *RCAC* Reproductive Concerns after Cancer Scale, *GAD-7* Generalized Anxiety Disorder-7.

The mean ages of the participants in the included studies ranged from 14.46 to 40.21 years. Most of the studies (*n* = 16, 64%)^28,29,32,33,35,36,39,40,44–46,48–50,53,55^ included cancer patients currently in young adulthood. Twenty-one studies (84%)^28–30,32–34,36–41,43–47,49,53,55,57^ included patients with various cancer diagnoses, while 4 studies included patients with breast cancer^35,48^, leukemia^51^ and lymphoma^50^. Twelve studies (48%)^29,30,35,36,41,43,44,47,50,51,53,55^ reported participants’ cancer treatment history. Ten studies (40%)^28,33,35,36,45–48,53,55^ recruited patients after active treatment (“survivors”), four studies (16%)^32,34,38,51^ recruited patients during treatment (“patients”), 10 studies (40%)^29,30,37,39–41,43,44,50,57^ recruited both patients and survivors, and one study (4%)^49^ recruited AYAs with advanced cancer (Table 2).

**Table 2:** Baseline characteristics of adolescents and young adults with cancer

Author, year	Country	Demographics					Clinical details									
		Mean age, years (SD), range	Female (%)	Education, *n* (%)	Employment, *n* (%)	Insurance, *n* (%)	Time since diagnosis, year	Cancer type, n (%)	Completed treatment	Chemotherapy	Radiotherapy	Surgery	Endocrine	Immunotherapy	Targeted	Transplant
Beale et al. ^27^ & Kato et al.^38^	Netherlands	15.92 (2.75), range: 13–29	32.3	Some college or more: 66 (17.8)	NA	NA	1.56 (2.4)	Blood cancer: 180 (48.5) Lymphoma: 61 (16.4) Brain tumor: 28 (7.6) Sarcoma: 61 (16.4) Other: 41 (11.1)	Ongoing							
Berg et al.^28^	USA	32.55 (5.45), range: 18–40	75	Bachelor or more: 27 (48.2)	Full- or part-time work: 39 (64.3) Student: 8 (14.3) Other: 12 (21.4)	53 (94.5)	2.07 (0.77)	Breast: 16 (28.6) Melanoma: 9 (16.1) Leukemia/lymphoma: 7 (12.5) Sarcoma: 5 (8.9) Colorectal: 3 (5.4) Testicular: 3 (5.4) Cervical: 2 (3.6) Other: 11 (19.6)	Completed							
Bergstrom et al. ^29^	Sweden	IG: 34.3 CG: 34.7 range: 19–40	84.1	University degree: 51 (65.9)	Employed/student: 115 (83.3)	NA	1.5	Breast: 66 (47.8) Cervical: 33 (23.9) Ovarian: 1 (0.7) Testicular: 14 (10.1) Lymphoma: 15 (10.9) Brain tumor: 9 (6.5)	Completed/ongoing	✓	✓		✓			
Breaky et al. ^30^	Canada	15.2 (1.7), range: 12–18	43.4	NA	NA	NA	15.2 (25.8) mos	Leukemia: 27 (36.4) Solid tumors: 12 (16.2) Lymphomas: 23 (31.1) Brain tumor: 5 (6.7) Other: 7 (9.4)	Ongoing: 65 (80.2) Completed: 9 (11.1) Unknown: 7 (8.6)	✓	✓	✓				✓
Ehrbar et al.^31,32^	Switzerland	29.31 (4.57), range: 18–40	100	University degree: 20 (39.2)	NA	NA	NA	Breast: 27 (53.0) Lymphoma: 14 (27.4) Astrocytoma: 4 (7.8) Gynecological: 4 (7.8) Colon: 2 (3.9)	Ongoing							
Greer et al.^33^	USA	25 (2.9), range: 19–29	80	some college degree or more: 38 (84.4)	NA	NA	2.7 (2.0)	Unclear	Completed							
Huyghe et al.^34^	France	IG: 32.4 (8.6) CG: 32.9 (10.4) range: 14–45	0	College degree or more: 10 (50.0)	NA	NA	NA	Testicular: 4 (20.0) Blood cancer: 8 (40.0) Other: 8 (40.0)	Ongoing							
Jiang et al.^35^	China	40.21 (4.24), range: 18–45	100	College degree or more: 57 (49.6)	Employed:78 (67.8)	NA	IG: 1.31 (0.26) CG: 1.53 (0.34)	Breast cancer	Completed	✓	✓	✓	✓			
Johnson et al.^36^	USA	33.6 (4.9), range: 18–39	55.1	Associate’s/bachelor’s degree or more: 23 (47.0)	Employed: 35 (71.4) Student: 3 (6.1)	44 (89.8)	3.6 (1.2)	Brain tumor: 9 (18.4) Breast: 6 (12.2) Lymphoma: 11 (22.4) Leukemia: 4 (8.2) Other: 19 (38.8)	Completed	✓	✓	✓				
Jones et al.^37^	USA	14.8 (1.96), range: 12–18	36.9	NA	NA	NA	NA	Unclear	Ongoing: 33 (50.8) Completed: 32 (49.2)							
Kirchhoff et al.^39^	USA	IG: 28.9 (7.2) CG: 28.7 (6.2) range: 18–39	68.6	College or more: 39 (45.3)	NA	100	IG: 6.5 (3.0) mos CG: 5.8 (3.4) mos	Unclear	Ongoing: 60 (69.8) Completed: 26 (30.2)							
Lang et al.^40^	Canada	IG: 28.9 (4.3) CG: 29.8 (5.8) range: 21–35	67.6	Postsecondary education: 23 (88.5)	Employed: 12 (46.2)	NA	IG: 7.5 (4.0–11.3) mos CG: 8.0 (5.0–17.0) mos	Hematopoietic and reticuloendothelial systems: 11 (42.3) Breast: 5 (19.2) Melanoma: 2 (7.7) Eye, brain tumor: 4 (15.4) Genital organs: 3 (11.5) Digestive organs: 1 (3.8)	Ongoing: 15 (57.7) Completed: 9 (42.3)							
Li et al.^42,43^	China	IG: 28.40 (6.275) CG: 31.21 (5.539) range: 15–39	71.2	Junior college/university degree or more: 22 (23.2)	NA	NA	<1 year: 73 (76.8) 1–3 years: 14 (14.7) >3 years: 7 (7.4)	Lung: 27 (28.4) Gynecological: 20 (21.1) Breast: 28 (29.5) Other: 20 (21.1)	Ongoing/completed	✓	✓	✓				
Li et al. ^41^	China	IG: 30.6 (6.3) CG: 29.7 (6.1) range: 15–39	73.3	Junior college/university degree or more: 35 (38.9)	NA	NA	<1 year: 69 (76.7) 1–3 years: 12 (13.3) >3 years: 9 (1.0)	Gynecological and breast: 29 (32.2) Gastrointestinal: 8 (8.9) Hematological: 31 (34.4) Head and neck: 19 (21.1) Other: 11 (12.2)	Ongoing/completed	✓	✓	✓				
Micaux et al.^44^	Sweden	IG: median 33 (range: 20–41) CG: median 34 (range: 19–40)	80.6	University: 73 (58.9)	Full- or part-time work: 86 (69.4) Student: 8 (6.5)	NA	1.5	Breast: 52 (41.9) Brain tumor: 14 (11.3) Cervical: 22 (17.7) Lymphoma: 19 (15.3) Ovarian: 5 (4.0) Testicular: 12 (9.7)	Ongoing: 45 (36.3) Completed: 79 (63.7)	✓	✓		✓			
Rabin et al.^45^	USA	32.17 (5.58), range: 18–39	55.6	Some college or more: 17 (94.44)	Full-time work: 12 (66.67)	NA	4.03 (2.52)	Testicular: 4 (22.2) Thyroid: 4 (22.2) Leukemia/lymphoma: 4 (22.2) Breast: 3 (16.7) Other: 3 (16.7)	Completed							
Rabin et al.^46^	USA	33.6 (4.0), range: 18–39	82.9	NA	Full- or part-time work: 25 (71.4)	NA	2.5 (2.1)	Unclear	Completed							
Sansom-Daly et al.^47^	Australia	20.6 (3.0), range: 15–26	52	University degree: 5 (16.7)	Full- or part-time work: 14 (46.7) Student: 9 (30.0)	NA	Age at diagnosis: IG: 17.8 (2.4), 13–23 CG: 19.4 (4.0), 11-25	Blood cancer: 16 (53.3) Solid tumor: 13 (43.3) Brain tumor: 1 (3.3)	Completed	✓	✓	✓				
Shin-Cho et al.^48^	USA	35 (3.3), range: 18–39	100	College degree or more: 22 (88.0)	NA	NA	<1 year: 1 (4.0) 1–3 years: 24 (96.0)	Breast cancer	Completed							
Snaman et al.^49^	USA	33 (6.1), range: 18–39	60	Some college degree or more: 47 (94)	NA	NA	NA	CNS tumor: 18 (36) Blood cancer: 4 (8) Solid tumor: 28 (56)	Ongoing							
Tock et al.^50^	Canada	32.35 (5.82), range: 20–39	84.6	Some college degree or more: 25 (96.2)	Full- or part-time work: 15 (57.7)	NA	NA	Lymphoma	Ongoing: 9 (34.6) Completed: 17 (65.4)	✓						
Uluhan et al.^51^	Turkey	IG: 14.7 (1.54) CG: 14.2 (1.28) range: 13–17	50	NA	NA	NA	NA	Leukemia	Ongoing	✓						
Valle et al.^55,56^ & 2017	USA	IG: 30.8 (5.7) CG: 32.7 (4.2) range: 21–39	90.7	College degree or more: 67 (77.9)	Full- or part-time work: 53 (61.6) Student: 17 (19.8)	NA	IG: 63.2 (7.8) mos CG: 53.7 (5.1) mos	Blood cancer: 27 (31.4) Breast: 17 (19.8) Gynecologic: 13 (15.1) Head and neck: 10 (11.6) Gastrointestinal: 9 (10.5) Other: 10 (11.6)	Completed	✓	✓	✓				✓
Valle et al, 2023a & 2023b & 2024	USA	33.40 (4.80), range: 18–39	81.8	College degree or more: 200 (71.4)	NA	260 (92.9)	3.66 (2.41)	Breast: 63 (22.5) Colorectal: 10 (3.6) Gynecological: 40 (13.9) Blood cancer: 15 (5.4) Lymphoma: 49 (17.5) Melanoma: 27 (9.6) Testicular: 16 (5.7) Thyroid: 30 (10.7) Other: 30 (10.7)	Completed	✓	✓	✓	✓	✓		✓
Zhang et al.^57^	USA	20.24 (2.17), range: 15–26	70.6	NA	NA	NA	NA	Leukemia: 8 (47.1) Lymphoma: 4 (23.5) Sarcoma: 4 (23.5) Giant cell tumor: 1 (5.9)	Ongoing: 10 (58.8) Completed: 7 (41.2)							

*SD* standard deviation, *NA* not available, *mos* months, *IG* intervention group, *CG* control group, *CNS* central nervous system.

Intervention durations ranged from 1^32,33^ to 5 months^39^. About half of the studies (*n* = 12, 48%)^28,29,32,33,40,41,43,44,46,47,49,53^ included post-intervention follow-ups, with approximately half of those having a follow-up period of around 3 months^31^. Nine studies (36%)^29,32,39,41,43,44,49–51^ provided a control group with usual care, 10 studies (40%)^28,30,35,36,38,45,48,53,55,57^ offered active control (*n* = 1)^57^ or attention placebo control (*n* = 9), five studies (20%)^33,34,40,46,47^ used waitlists, and one study (4%) provided printed materials.

### Overview of digital health interventions

Details of the DHIs in the included studies are summarized in Table 1. Thirteen studies (52%)^29,30,32,34,38–40,44,45,48,51,52,55^ used a website or web-based application. Six studies (24%)^36,43,46,50,53,55^ used wearable electronic devices, five^36,43,50,53,55^ of which were accompanied by a smartphone app. Six studies (24%)^28,41,46,47,50,57^ delivered interventions via videoconferencing or telephone. Five interventions (20%)^28,33,35,53,55^ were conducted via specially designed mobile applications. Eight studies (32%)^36,38,43,46,50,51,53,55^ provided participants with the required technology and devices.

The contents of the majority of the interventions were multicomponent and promoted self-management, which were classified into informational (cancer-related information, psychoeducational modules), skill-based (self-management training, behavioral skills training), psychotherapeutic (cognitive and behavioral training, mindfulness-based practices, optimism training), social (peer support), and lifestyle (personalized physical exercise) domains.

Most of the interventions included interactive features, such as videos (*n* = 15, 60%)^28–30,33–35,37,38,40,44,49,51,53,55,57^, online chat groups or discussion forums (*n* = 12, 48%)^29,35,36,40,41,43–46,50,53,55^, telephone consultations (*n* = 7, 28%)^28,30,35,43,46,53,57^, and instant messaging (*n* = 5, 20%)^28,35,36,53,55^ with health professionals. Twelve interventions (48%)^28,30,35,36,43,46–48,50,53,55,57^ involved repeated contact with research staff or healthcare professionals. Eleven interventions (44%)^28,36,39–41,43,46–48,50,57^ were primarily instructor-led, while 14 (56%)^29,30,32–35,37,38,44,45,49,51,53,55^ were mainly self-guided.

### Risk of bias

**Fig. 2:**
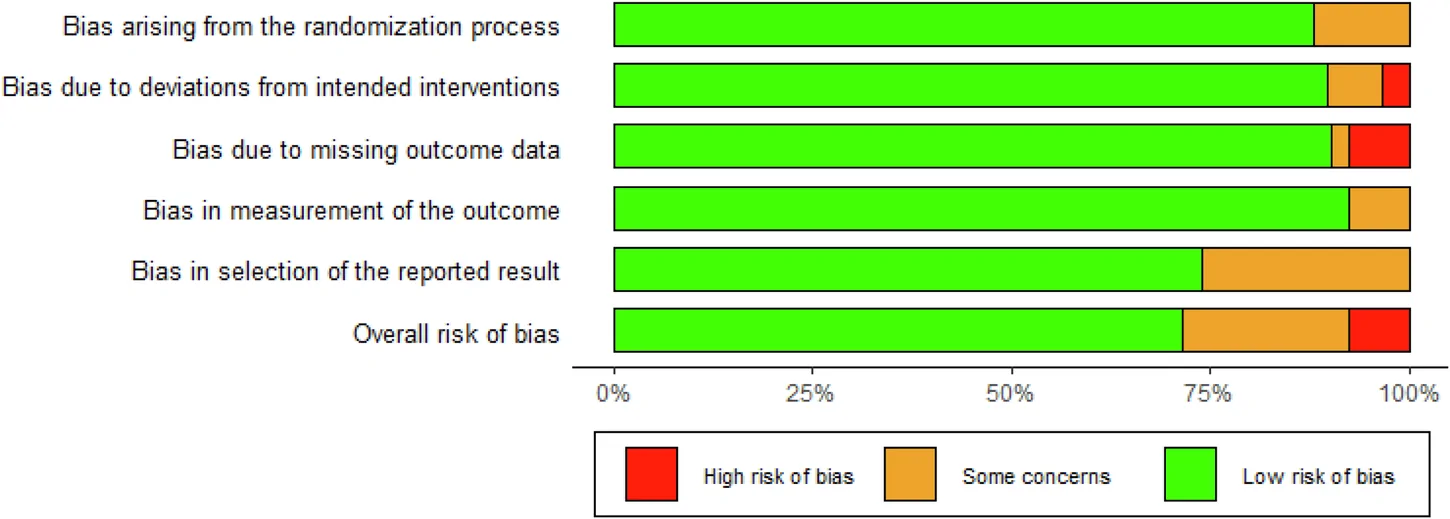
Summary of risk of bias across bias domains for included studies. The graph displays the authors’ judgment on the risk of bias of included studies, presented as percentage totals according to the Cochrane Collaboration Risk of Bias 2 Tool.

**Fig. 3:**
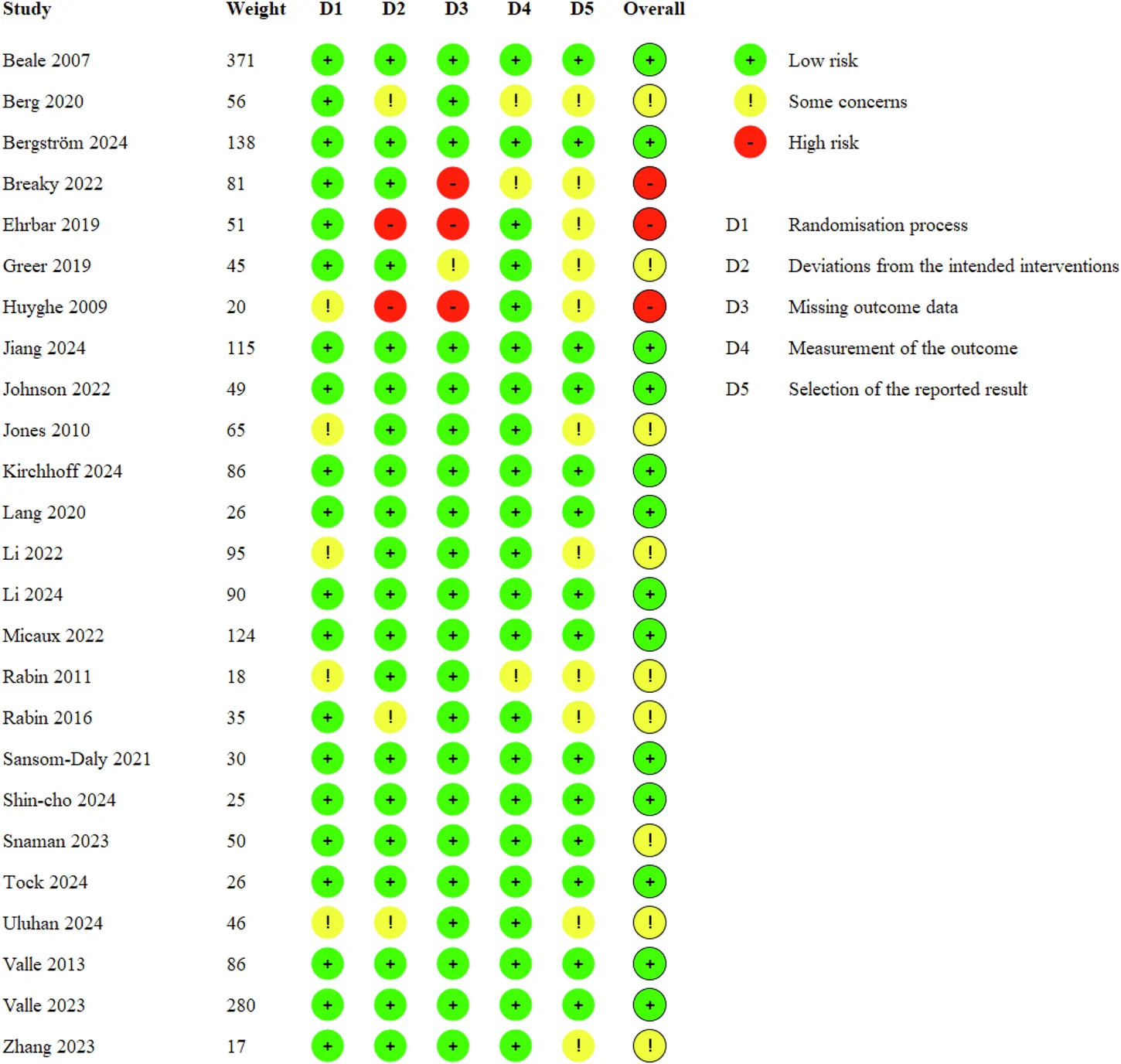
Graphical visualization of the risk of bias results for each included study.

Figures 2 and 3 provide summaries of the risk of bias (RoB) among the included studies in different domains. Low RoB was identified in 13 studies^29,35,36,38–41,44,47,48,50,53,55^ across all domains of the RoB2 tool. The RoB2 assessment revealed “some concerns” in nine trials^28,33,37,43,45,46,49,51,57^, primarily related to the unavailability of the study protocol or histories of trial registry (8 of 9), followed by insufficient details regarding the randomization process (4 of 9). In most of the included studies, the participants and interventionists were not blinded to the intervention assignment because of the nature of behavioral interventions. High attrition rates were observed in two trials^30,32^, and appropriate methods for handling missing data (e.g., sensitivity analysis) were not used, leading to a high RoB in two studies. One study was assessed as having a high RoB because of a lack of information about participant attrition and the analysis methods used to treat missing outcomes^34^. Assessment of the funnel plots and combined Egger’s tests revealed no significant publication bias.

### Quality of life

**Fig. 4:**
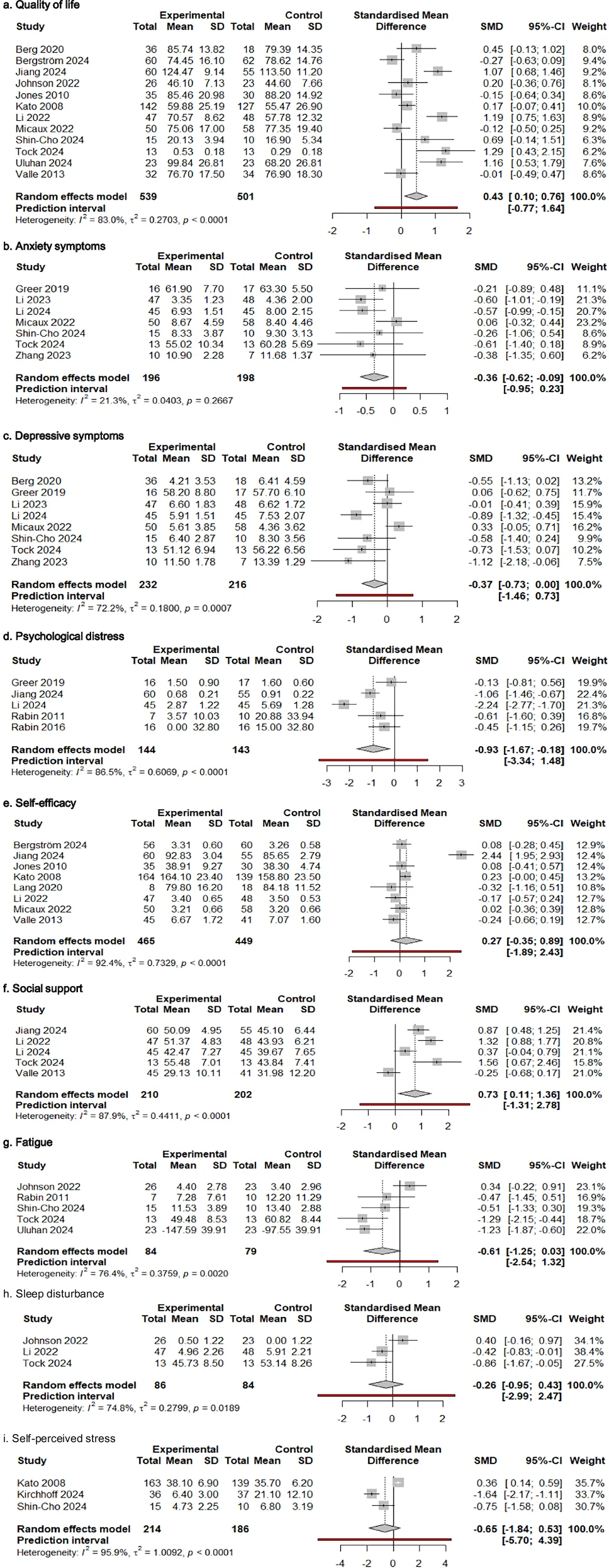
Forest plot for effects of digital health interventions on psychological outcomes. This figure shows the pooled effect of digital health intervention on **a** quality of life, **b** anxiety symptoms, **c** depressive symptoms, **d** psychological distress, **e** self-efficacy, **f** social support, **g** fatigue, **h** sleep disturbance, and **i** self-perceived stress. SD standard deviation, SMD standard mean difference.

The effect of DHIs on QoL was measured in 15 studies^28–30,35–38,43,44,47,48,50,51,54,55^, with 10 (66.7%)^28,35,36,43,48,50,53^ involving repeated contact with health professionals or research staff. Fourteen studies (93.3%) had low to moderate RoB. Direct comparison data for QoL were unavailable for three studies^30,47,54^. A meta-analysis excluding these studies (*n* = 12) demonstrated a significant standardized mean difference (SMD) of 0.43 (95% confidence interval [CI]: 0.10–0.76) favoring DHIs in terms of increasing QoL, although heterogeneity was high (*τ*^2^ = 0.27, *I*^2^ = 83.0%, *p* < 0.01; Fig. 4a). Mean age, patient type, intervention period, condition of the control group, mode of intervention delivery, and level of RoB were not significant moderators (Supplementary Table 1).

### Anxiety symptoms

Anxiety symptoms were measured in 11 studies^29,30,33,40–42,44,47,48,50,57^, seven (63.6%) of which^28,30,42,47,48,50,57^ involved repeated contact with health professionals or research staff. After the exclusion of four studies^29,30,40,47^ without direct comparison data, the meta-analysis revealed a significant reduction in anxiety (*n* = 7; Fig. 4b), with an SMD of -0.36 (95% CI: −0.62 to −0.09). Heterogeneity was low to moderate (*τ*^2^ = 0.04, *I*^2^ = 21.3%, *p* = 0.27). The mode of intervention delivery was a significant moderator, as studies^28,41,42,48,50,57^ with instructor-led interventions were more likely to result in reduced anxiety symptoms than those^33,44^ with self-guided interventions. Mean age, patient type, condition of the control group, and level of RoB were not significant moderators. The intervention period was a marginally significant moderator of anxiety symptoms (Supplementary Table 1).

### Depressive symptoms

Depressive symptoms were measured in 12 studies^28–30,33,40–42,44,47,48,50,57^. Eleven studies (91.7%) had low to moderate RoB. After the exclusion of four studies^29,30,40,47^ without direct comparison data, the meta-analysis revealed no statistically significant reduction in depressive symptoms (*n* = 8; Fig. 4c). Heterogeneity was low to moderate (*τ*^2^ = 0.18, *I*^2^ = 72.2%, *p* < 0.01). However, upon removal of the study by Micaux et al.^44^, the meta-analysis yielded statistically significant results (SMD = -0.490, *p* = 0.039). The mode of intervention delivery was also a significant moderator of the efficacy of DHIs on depressive symptoms, while mean age, patient type, condition of the control group, and level of RoB were not (Supplementary Table 1).

### Psychological distress

Five studies^33,35,41,45,46^ measured psychological distress, with two^35,41^ reporting significant intervention effects. The meta-analysis revealed a significant reduction in distress favoring DHIs, with an SMD of −0.93 (95% CI: −1.67 to −0.18). However, high heterogeneity was found between studies (τ^2^ = 0.61, *I*^2^ = 86.5%, *p* < 0.01; Fig. 4d). Studies with low RoB and usual care as the control showed greater reductions in the level of distress compared with those having some concerns of bias and waitlist controls. Mean age, patient type, intervention delivery mode, and duration were not significant moderators (Supplementary Table 1).

### Self-efficacy and social support

Self-efficacy was measured in eight studies^29,35,37,38,40,43,44,55^, and social support was assessed in five studies^35,41,43,50,55^. The meta-analysis found no significant improvement in self-efficacy (Fig. 4e) but a significant increase in social support (SMD 0.73 [95% CI: 0.11–1.36]; Fig. 4f). Heterogeneity was high for both outcomes (self-efficacy: *τ*^2^ = 0.73, *I*^2^ = 92.4%, *p* < 0.01; social support: *τ*^2^ = 0.44, *I*^2^ = 87.9%, *p* < 0.01). Mean age, patient type, condition of the control group, intervention period, mode of intervention delivery, and level of RoB were not significant moderators. Moderator analysis was not performed for subgroups with only one study (Supplementary Table 1).

### Fatigue

Fatigue was measured in five studies^36,45,48,50,51^. The meta-analysis revealed a marginally significant reduction in fatigue (SMD −0.61 [95% CI: −1.25 to 0.03]), with high heterogeneity between studies (*τ*^2^ = 0.38, *I*^2^ = 76.4%, *p* < 0.01; Fig. 4g). In the sensitivity analysis, after the exclusion of Johnson et al. study^36^, the intervention effect became significant (SMD −0.94 [95% CI: −1.37 to −0.51]) and heterogeneity decreased between the remaining studies (*τ*^2^ = 0.03, *I*^2^ = 12.1%, *p* < 0.01). The type of control group was a significant moderator, with studies using usual care^50,51^ as the comparator showing greater fatigue reduction than those with active/attention placebo controls performing other targeted interventions without the digital or multimodal aspect/component^36,45,48^. Mean age, delivery mode, and level of RoB were not significant moderators (Supplementary Table 1).

### Sleep disturbance

Sleep disturbance was measured in three studies^36,43,50^, all of which implemented online exercise programs incorporating wearable electronic devices and companion mobile applications. All three studies included repeated contact with health professionals or research staff. The meta-analysis demonstrated no significant improvement in sleep quality (SMD −0.26 [95% CI: −0.95 to 0.43], Fig. 4h).

### Self-perceived stress

Perceived stress was evaluated in three studies^38,39,48^, with two interventions being instructor-led and having usual care as the comparator. All of the studies used websites to conduct interventions, and all were rated as having low RoB. The meta-analysis demonstrated no significant improvement in the reduction in perceived stress (SMD −0.65 [95% CI: −1.84 to 0.53], Fig. 4i).

### Physical activity

Physical activity was measured in seven studies^36,42,45,46,50,55,57^, all of which incorporated chat groups or discussion forums to facilitate peer support among participants. Six studies (85.7%)^36,42,46,50,53,55^ included repeated contact with health professionals or research staff, as well as the use of wearable electronic devices. Objective physical activity levels were measured using Actigraph GT3X+ devices in two studies^36,53^, while self-reported activity levels were measured in six studies^42,45,46,50,55,57^ using validated measures: the International Physical Activity Questionnaire, Seven-Day Physical Activity Recall, and Godin Leisure Time Exercise Questionnaire. Two studies^36,53^ also reported the participants’ sedentary behavior. Three studies^42,50,59^ in which members of the control group received usual care or were waitlisted showed significant intervention effects on total physical activity level or moderate-to-vigorous physical activity. In contrast, DHIs did not have a statistically significant effect on physical activity compared with control groups receiving attention placebo or active-control interventions^36,45,53,55^. Meta-analysis was not performed for physical activity because of high heterogeneity in the outcomes measured across studies.

### Other outcomes

Additional outcomes assessed across the studies included body image^50^, reproductive concerns^44^, sexual dysfunction^29^, financial distress and health insurance literacy^39^, post-traumatic growth^40^, fear of cancer recurrence^48^, and decisional conflict^32,49^.

One study^47^ evaluated the health economics of DHIs, demonstrating that compared with an online peer-support group, participants receiving online group-based psychological support (“Recapture Life”) reported higher health service utilization (*p* = 0.05) at 6–12 months post-intervention, more frequent visits to their general practitioner at the 12-month follow-up (*p* = 0.015), and increased emergency department visits at the 12-month follow-up. However, no differences were observed in mental health service use and hospital admission between the two groups over time. In terms of financial outcomes, the cost of delivery in “Recapture Life” (AU$485–540 per participant) was higher than that of the online peer-support group (AU$365–415 per participant). Participants receiving DHIs in this study avoided AU$260 in travel costs regarding fuel costs alone.

### Acceptability

Sixteen studies (64%)^27–30,32–34,37,39,40,45,46,48,50,55,57^ evaluated participants’ perceived acceptability of the interventions. Most participants found the psychoeducational websites and personalized exercise programs acceptable (satisfaction: 60.5–100%^27,29,30,34,45,46,48,50^), helpful or useful (42.9–92.3%^34,45,48,50^), and easy to use or understand (86–100%^34,37,45^). The majority expressed a willingness to recommend the programs to other similar cancer patients (80–100%^28,29,32,34,39,45,50^). However, in one study^55^, only 46.8% of the participants in the intervention group stated that they would recommend the program to others, compared with 61.8% in the active control group (61.8%, *p* = 0.225). In another study^30^, some participants receiving a 12-week web-based self-management program reported feeling overwhelmed and anxious about completing 12 modules, which may have contributed to the high attrition rate (66.7%). More details are shown in Table 3.

**Table 3:** Summary of process evaluation data in included studies

Author	*n* Most complex intervention	Intervention type	Intervention length	Acceptability	Engagement
Beale et al.^27^ & Kato et al.^38^	195	Website: Re-Mission	3 mos	About 60/195 chose “agree” for the rating of acceptability, and about 58/195 chose “strongly agree” for the rating of acceptability	Time using study computers: 10.7 h (SD 1.0), 33% used computers for the requested duration of at least 1 h per week 16.4% (32/195) did not play assigned game(s) at all
Berg et al.^28^	38	1. Smartphone App: Achieving Wellness After Kancer in Early life (AWAKE) app 2. Telephone-based coaching	2 mos	94.3% recommend keeping the coaching, and over 80% recommend keeping the behavior/mood tracking and homework. Over 88% would recommend the program to other cancer survivors. Most considered the video relevant: mean 3.14 (SD 1.00) (range: 0“not at all” to 4“very”)	Completed 6.16/8 (SD 2.83) coaching sessions, watched 6.79/8 (SD 2.00) weekly videos, completed 4.50/8 (SD 2.31) weekly homework assignments, mean 6.92/8 (SD 1.92) weeks completing at least one intervention component. Mean watched 3.64 (0.93) video content (range: 1“none” to 4“most or all”)
Bergstrom et al.^29^	72	Website: Fex-Can Sex program	3 mos	73% (41/56) said that they appreciated the content of the program 91% (51/56) would recommend the program to others	Only 16/72 (22%) were categorized as high-activity users, 40/72 (56%) opened three modules or more, 19/72 (24%) responded to at least 50% of the reflective questions and the quiz, and 5 (6.9%) never opened the program. Time spent in the program: mean 20.7 (SD 21.3) minutes Time spent in the discussion forum: mean 4.0 (SD 9.0) minutes Only 7/72 (9.7%) wrote their own posts in the forum.
Breaky et al.^30^	39	Website: self-management program + monthly calls from health coaches	3 mos	Satisfaction: 81.5% (13/16) completed the adolescent satisfaction questionnaire, agreeing or strongly agreeing that the program was helpful Some felt that completing the modules could feel overwhelming and could be anxiety-provoking for youth.	46.2% (18/39) logged into the website, 28.2% (11/39) completed at least one module, a mean of 2.4 modules completed (Median 1.0, SD 3.0, range 0–9), none completed the full program
Ehrbar et al.^31,32^	24	Website: online decision aid (DA)	1 mos	Website satisfaction: mean score 3.79/5 (SD 0.72) Perceived helpful: mean score 3.54/5 (SD 0.78) 80% would recommend to others: mean score 3.96 (SD 0.62)	Mean accessed the DA: 2.23 times (range: 1–8), mean time per session: 9 min 10 s, visited an average of 8.96 pages per session. 45% conducted in the information part, and 40% in the clarification exercises. 80% completed the value clarification exercises
Greer et al.^33^	25	Chatbot: *Vivibot*	1 mos	Helpful: mean score 2.03/3 (SD 0.73, range: 0–3) Likely to be recommended to friends: 6.9/10 (SD 2.6, range: 0–10)	Spent an average of 73.8 min (SD 52) across an average of 12.1 (SD 7.1) engaged sessions chatting with *Vivibot*
Huyghe et al.^34^	10	Website: Banking on Fatherhood (BOF)	NR	Easy to understand: 100%; including enough information: 90%; boring: 10%; valuable: 95%; personal stories useful: 80%; information relevant: 85%; valuable to other similar patients: 100%	NR
Jiang et al.^35^	60	1. Mobile App: AI-TA 2. Web-based follow-up	3 mos	NR	NR
Johnson et al.^36^	26	Fitness tracker: Fitbit Flex + Fitbit app + Facebook group + PA “buddy”	3 mos	NR	Eligible days wore the Fitbit: 82.9% 22/26 (84.6%) joined the private Facebook group Number of group posts: median 61.5 (IQR: 50.0–66.0); liking group wall posts: median 7 (IQR: 1.0–14.0); comments: median 2.0 (IQR: 0.0–10.0); posts: median 0.0 (IQR: 0.0–1.0) 21/26 (80.8%) selected a PA “puddy”
Jones et al.^37^	35	Multimedia CD-ROM	3 mos	Easy to use: 91.4%	7/35 (21.2%) used the CD-ROM many times; 14/35 (45.5%) used the CD-ROM a few times
Kirchhoff et al.^39^	48	Website: HIAYA CHAT	5 mos	Satisfaction (range: 9–45): mean score 37.5 (SD 7.8) Among those exited interviews content areas, thought the intervention important or would recommend: 10/10 (100%), enjoyed the intervention: 10/10 (100%), endorsed Zoom format: 9 (90.0%)	29/43 (67.4%) completed all four navigator sessions, 11/43 (25.5%) did not complete any sessions or declined after random assignment, 5/43 (11.7%) completed one or two sessions
Lang et al.^40^	8	Website: *cancerchatcanada.ca*, CCC	2.5 mos	Easy to use (system usability scale, range: 0–100): mean score 88.0 (SD 9.7)	60% of participants attended 7 or more sessions
Li et al.^42,43^	47	Wearable electronic device + WeChat group	2 mos	NR	NR
Li et al.^41^	45	Online-based peer support	2 mos	NR	NR
Micaux et al.^44^	64	Website: Fex-Can Fertility program	3 mos	NR	21/64 (33%) were categorized as high-activity users, 33/64 (51%) were low-activity users, and 10 (16%) never logged onto the website. 11/64 (17%) made at least 1 posting and 20/64 (31%) actively read the posts for 3 min or more
Rabin et al.^46^	8	Website: adapted PA website based on the Step into Motion website	3 mos	86% of participants would recommend to other Satisfaction: 71% Very easy-to-understand website information: 86% Helpful: 42.9–71.5% 43% reported no barriers to log onto the website, some reported lack of time, work commitment, slow internet connection	Number of website logins: mean 14.75 (SD 8.46) Number of days on PA logging: mean 11.38 (SD 7.93) Number of days on Goal setting: mean 5.25 (SD 4.17) Number of days on Stage-based manual: mean 3.13 (SD 2.17) Number of days on PA-related information: mean 1.25 (SD 1.28) Number of days on PA resources: mean 0.88 (SD 1.13) Number of days on PA tips: mean 0.50 (SD 1.07) Reasons for interfering with meeting PA goals: lack of time, work/family responsibilities
Rabin et al.^46^	19	Telephone-based PA intervention + activity tracker + CD-based meditation intervention	3 mos	96% of participants said the length of weekly calls was “just right”, 71% of participants felt that the weekly PA goals were just the right amount of time and 64% felt the right amount of time for meditation Satisfaction: 97% gave 4 or 5 ratings out of 5 for PA component and 61% for the meditation component All of those completing the participant evaluation said would recommend it to others	NR
Sansom-Daly et al.^47^	19	Videoconference: Recapture Life intervention	1.5 mos	NR	NR
Shin-Cho et al.^48^	15	Online expressive writing	1.5 mos	Satisfaction: 100% said enjoyable Effective in reducing stress levels: 72%	Completed at least one writing task: 13/15 (87%), two writing tasks: 11/15 (73%), three writing tasks: 5/15 (33%) Reason for not completing task: being busy, forgetting
Snaman et al.^49^	25	Video: Advance care planning video	NR	NR	NR
Tock et al.^50^	13	Videoconference + Fitbit app + activity tracker + discussion with a kinesiologist	3 mos	84.6% (11/13) thought personalized exercise programs were helpful in motivating to exercise 84.6% (11/13) were satisfied with the remote format of the exercise program 76.9% (10/13) felt the frequency of the kinesiologist follow-up meetings was acceptable 92.3% (12/13) thought wearing the Fitbit and receiving an alert to motivate them to exercise was helpful 53.8% (7/13) enjoyed using the peer support group on the app 100% (13/13) thought the time completing the program was acceptable 92.3% (12/13) would recommend the program to others	Valid Fitbit wear days over the 12-week: 90% (982/1092)
Uluhan et al.^51^	23	Website: Re-Mission	3 mos	NR	NR
Valle et al.^55,56^ & 2017	45	Facebook-based intervention (FITNET): behavioral lessons via Facebook + Activity tracker + mobile app + weekly message + website + PA diary + Facebook group discussion	3 mos	Very easy to access study information (range: 1“strongly disagree” to 7“strongly agree”): 5.1 ± 1.4 Thought accessing study information was effective in getting information about exercise (range: 1“strongly disagree” to 7“strongly agree”): 4.9 ± 1.4 Enjoyable (range: 1“strongly disagree” to 7“strongly agree”): 4.6 ± 1.7 46.9% would recommend the program to others	81.3% reported receiving 10 or more messages, 62.5% reported usually reading some to all/most of the Facebook messages Used various Facebook group features 1–2 days a week (range: 1–6 “several times a day”): visited the Facebook group (2.6 ± 1.3); saw a FITNET group post in the News Feed (2.8 ± 1.0); read FITNET group discussions (2.7 ± 1.1) Posted 153 Facebook comments to the group wall over 12-week (mean number of posts: 3.4 ± 4.6), 48.9% (22/45) made 2 or more Facebook posts Setting goals: 4.2 ± 4.8 goals (range: 0–13) over 12 weeks, 30/45 (66.7%) used the goal-setting feature at least once Submitted mean PA entries: 21.9 ± 37.9 (range: 0–170) and mean steps entries: 13.1 ± 24.2 (range: 0–78) over the 12-week and 32/45 (71.1%) tracked any exercise data at least once Steps entries declined from 1.3 ± 2.2 to 0.7 ± 1.7 over 12 weeks, logging declined from 57.8% in week 1 to 24.4% in week 12
Valle et al.^52,53^ & 2024	140	Mobile PA intervention: Improving Physical Activity after Cancer Treatment (IMPACT)	3 mos	NR	Median days of using Fitbit activity: 174/182 days [Mdn(IQR: 96% (83%, 99%)] Median weeks of engaging within the Facebook group: 11/26 weeks [Mdn (IQR: 42% (23%, 62%)] Median weeks of login onto the website 1 time or more: 23/26 weeks [Mdn (IQR: 88.5% (63.5%, 100%)] Average time of having 7 days of accelerometer wear: 15.4 h/day at baseline and 15.5 h/day at 6 months
Zhang et al.^57^	10	Telephone or web-based videoconference intervention: Mind Your Total Health (MYTH)	2 mos	Acceptability of intervention measure (AIM, range: 1“completely disagree” to 5“completely agree”): mean 4.69 (SD 0.44)	8/10 (80%) completed at least 6 out of 8 sessions, 6 completed all sessions within 10 weeks, and 2 completed 6 out of 8 sessions within 10 weeks

*SD* standard deviation, *Mdn* median, *IQR* interquartile range, *mos* months, *NR* not report, *PA* physical activity.

### Engagement

Seventeen studies (68%)^27–30,32,33,36,37,39,40,44,45,48,50,53,55,57^ evaluated participant engagement with the interventions, measuring metrics such as usage tracking, module completion, time spent, and interaction participation. Most participants (46.2–93.1%^27,29,30,32,36,37,39,40,44,48,50,53,55^) engaged with the intervention at least once (e.g., logging in or opening modules). Studies^36,50,53^ incorporating wearable electronic devices reported high engagement, with participants using the devices on 82.9–96.0% of eligible wear days across the intervention. However, large proportions of participants (51–78%^27,29,30,44^) demonstrated low activity on the intervention websites. Two studies^48,55^ noted a decline in engagement over time. Common reasons for not continuing engaging the intervention included lack of time, forgetfulness, and competing family or work commitments^45,48^ (Table 3).

## Discussion

This is the first systematic review and meta-analysis examining the efficacy of DHIs in AYAs with cancer using evidence exclusively from RCTs. We found that DHIs effectively improved QoL, reduced anxiety and psychological distress symptoms, and enhanced social support but had no effect on self-efficacy, fatigue, sleep disturbance, or self-perceived stress. Delivery mode significantly moderated the effects of DHIs on anxiety and depressive symptoms, with instructor-based delivery producing stronger effects. However, the pooled effect on depressive symptoms remained non-significant. The results of the implementation outcomes revealed that DHIs were generally acceptable, helpful, and easy to use; however, attrition was common, especially for DHIs with complex modules. Few studies reported an economic evaluation of their interventions, and the RoB assessment highlighted the absence of study protocols or inadequate reporting of pre-specified outcomes.

Most of the RCTs (68%) were conducted in the last 5 years, reflecting the growing interest in and need for stronger evidence regarding these interventions. The scope of the psychological function targets of DHIs is becoming extensive, encompassing not only psychological distress but also areas that are particularly pertinent to AYAs, such as financial toxicity in oncology, sexual and reproductive health, and treatment decision-making. This breadth of scope demonstrates the potential of DHIs to improve outcomes across various dimensions of AYAs’ health and well-being. However, 84% of the studies included were conducted in high-income countries, and in the two studies reporting participants’ residence settings, more than two-thirds of the participants in the intervention group resided in urban areas. One review^60^ summarized the current evidence about rural–urban disparities in cancer outcomes and highlighted that research should prioritize attenuating these disparities. Because digital health tools have great potential to address such gaps, research should include AYAs living in rural areas or be conducted in low- and middle-income countries.

This review revealed that DHIs have significant positive effects on patient-reported outcomes (PROs), including QoL, anxiety and psychological distress symptoms, and social support, while variation across delivery modes may have diluted the pooled effect on depressive symptoms. Most of the interventions were multicomponent in nature. Hence, this review was unable to identify the effects attributable to each intervention component. Nevertheless, the observed components in this review may have different mechanisms for improving outcomes. To illustrate this point, we provided several examples of how the components of the intervention may affect the outcomes: (1) informational components may enhance AYAs’ knowledge of their illness and self-efficacy, thereby contributing to reductions in worries and distress and improvements in QoL. Such components are particularly effective in addressing age-specific informational needs related to fertility/sexuality, peer/family relationships, work/school, and financial concerns^61^, (2) skill-based and psychotherapeutic components may equip AYAs with coping and problem-solving skills to manage symptoms. Monitoring and tracking devices can facilitate symptom monitoring and self-regulation, enabling more personalized interventions and supporting adherence^62^, and (3) social components can directly enhance perceived social support and connectedness, reduce isolation, and increase engagement^41^. Besides critical intervention content, other aspects such as interpersonal support (e.g., contact with health professionals), factors affecting engagement and adherence, and presentation features (e.g., gamification) should also be regarded as an “overarching component”^63^. To identify active components of DHIs, future RCT design should consider a multiphase optimization strategy (MOST), which involves establishing a set of components that are candidates for inclusion, specifying an optimization criterion for the entire intervention, and then collecting experimental data to identify the subset of components that meet the criterion^64^.

We found that the instructor-led delivery mode was associated with greater effects on anxiety and depressive symptoms among AYAs than the self-guided mode, which is consistent with the findings of similar reviews^65,66^. Interventions that included repeated contact with a health professional or researcher or that had an AYA-specific social forum showed significant effects on PROs. These results suggest that providing interpersonal support in DHIs, even at a minimal level, can be a significant moderating factor influencing engagement, adherence, and outcomes by creating a supportive environment for AYAs and motivating their continued participation^67,68^. Importantly, the level of interpersonal support across DHIs varies in terms of the provider (e.g., researcher, clinician, or virtual human), the degree of support provided (e.g., semi-guided or fully guided), its purpose (e.g., to provide encouragement or to check for technical issues), and the uniqueness of the support (e.g., tailored or automated). Future RCTs should provide detailed reporting of these aspects, since the type and intensity of human support constitute key parameters for evaluating the cost-effectiveness of DHIs and determining their scalability in clinical practice.

DHIs did not significantly improve self-efficacy among AYAs, in contrast to the findings of a previous review^69^. Low engagement in half of the included interventions is likely to have reduced their impact. AYAs’ competing priorities, such as school, work, and social activities, limited their participation^45,48^. Although AYAs are often considered “digital natives,” complex interfaces can still hinder their engagement and reduce the interventions’ efficacy^30^. Our findings also indicated that DHIs did not have a significant effect on fatigue among AYAs; however, excluding the study by Johnson et al.^36^ yielded a significant overall effect. Moreover, moderator analysis revealed that DHIs significantly reduced fatigue in RCTs where the control group received usual care, but not in RCTs with active or attention placebo controls using targeted interventions without the digital component or multimodal components. A similar pattern was observed for physical activity outcomes, though a quantitative effect was not synthesized. Active controls are critical for distinguishing between digital engagement effects and intervention-specific mechanisms^70^. When both groups used some form of targeted intervention, the positive effects attributed to the additive effect of multiple components may have masked the unique benefits of DHIs alone. Future research should use a hybrid design, such as a three-arm trial, to disentangle intervention-specific effects from general digital management effects.

Engagement is an essential element for any DHIs; beyond usage and adherence, meaningful engagement should also be assessed across cognitive, emotional, and behavioral dimensions^71^. The majority of the participants in the studies included in this review perceived DHIs as acceptable, easy to use, and recommendable, indicating positive cognitive engagement that has a direct impact on subsequent behavioral engagement and an indirect impact via affective engagement, such as satisfaction with the interventions^39^. Conversely, high attrition was found in interventions with complex modules that require lots of attention, as participants often felt overwhelmed^30^. We also found a decreased use of interventions over time in two studies^48,55^, commonly due to lack of time, forgetfulness, and competing family or work commitments. Given that AYAs often experience time constraints, DHI design should include content in brief sessions or an adaptive feature that can adjust the content and intensity of the intervention according to users’ progress and feedback^72^.

Reminders that support cognitive engagement, as well as interactive features and rewards that foster affective and cognitive motivation, have been shown to enhance behavioral engagement and efficacy within DHIs^73^. However, behavioral engagement with the online forums and chat groups was not as high as expected in this review. This discrepancy may be explained by the fact that AYAs often prioritize security and privacy, and they may perceive public forums as unsafe for discussing sensitive topics (negative cognitive engagement), leading to superficial participation (e.g., passive lurking)^74,75^. The interactive feature within DHIs could also increase the attention burden of participants or make them feel demotivated by the social comparison or judgment within the digital community. This may trigger negative affective and/or cognitive disengagement, resulting in decreased behavioral engagement. To avoid this, interactive features need to be personalized, kept as flexible as possible, and offered as optional for AYAs. Of note, greater engagement does not necessarily correspond to improved clinical outcomes. Low engagement may reflect the use of skills beyond DHIs, reliance on alternative resources, or the achievement of recovery^76^. Researchers need to consider whether increased and sustained engagement is always required by assessing the “target dose” and reporting engagement trajectories. Although it remains unclear which factors contribute most effectively to active engagement and improved outcomes, co-design holds promise for improving both delivery and engagement^77^.

Despite providing a summary of evidence from RCTs and delineating the pooled effect sizes of DHIs on a comprehensive list of psychological outcomes relevant to survivors of AYA-onset cancer^5^, this systematic review has several limitations. First, 84% of the studies were conducted in high-income countries, focusing on urban-dwelling, highly educated AYAs. This may indicate that AYAs with limited access to digital services were not represented, and thus the results may not be generalizable to low- or middle-income countries or to AYAs living in rural areas or having lower education attainment. Second, variability in participants, intervention components, and outcome measurements across the included studies may have contributed to heterogeneity, underscoring the need to interpret the observed effects with caution. Given the heterogeneity of the intervention components, we did not evaluate the intervention-specific evidence synthesized from this review. In addition, follow-up times varied across studies. Because of the limited number of studies with follow-up periods beyond 6 months, we did not perform a meta-analysis to investigate the long-term efficacy of the DHIs for each outcome. Third, we initially intended to synthesize evidence on the cost-effectiveness of DHIs versus control groups, but we identified only one relevant study. Future RCTs should incorporate economic evaluations as an outcome to identify low-cost support features that could reduce rural–urban disparities in supportive care access.

In addition, cognitive impairment is a critical domain of psychological function for AYAs during their survivorship period^5^. This outcome is highly relevant to AYAs, who are in a critical period of brain development and reorganization. Observational studies have shown that cognitive deficits in AYAs may impact education, employment, and social participation, which may adversely affect their financial stability and decrease their quality of life^7^. Recent evidence has shown the positive effects of DHIs with cognitive training on cognitive impairment among older breast cancer patients^78^. One study has demonstrated the feasibility of a computer-based cognitive rehabilitation program in improving cognitive outcomes among AYAs with cancer^79^. However, there are still a few interventional studies specifically targeting cognitive outcomes in the AYA population, likely because cognitive impairment has only recently become a more explicit focus in oncology care, and the evidence base is still emerging. More RCTs with cognitive impairment as a primary outcome are therefore needed to evaluate the efficacy of DHIs in AYAs. There is also a need to address challenges such as low participation and retention rates, as well as the measurement burden faced by this population. Furthermore, one study demonstrated that self-reported and objective measures of cognitive functions may capture different constructs, and perceived cognitive difficulties may reflect psychological distress rather than actual cognitive impairment^80^. To strengthen the evidence base for intervention efficacy, both self-reported cognitive difficulties assessed with validated measurement tools and objective cognitive tests are recommended.

In conclusion, this systematic review with meta-analyses provides evidence that DHIs significantly improve QoL, reduce anxiety and psychological distress symptoms, and enhance social support compared with usual care. The effects on depressive symptoms and fatigue remain inconclusive, underscoring the need for more rigorous RCTs. Most of the interventions were multicomponent, interactive, self-management programs. Given AYAs’ preferences, future interventions should consider incorporating instructor-led delivery modes, artificial intelligence or wearable devices, simplified modules, and optional interactive features. Importantly, interventions that are co-designed with AYAs may improve engagement.

## Methods

This systematic review and meta-analysis adhere to the 2020 Preferred Reporting Items for Systematic Reviews and Meta-Analyses (PRISMA) guidelines. The study protocol has been registered in the international database of prospectively registered systematic reviews (PROSPERO; CRD42024593656).

### Search strategy and eligibility criteria

We conducted a comprehensive search in PubMed, Ovid Embase, EBSCO CINAHL Plus, The Cochrane Library, APA PsycINFO, and ProQuest from their inception to January 5, 2025. The references of included articles and similar systematic reviews were hand-searched to ensure that the search was exhaustive. Databases were searched using a combination of controlled search terms and free-text terms for digital health, adolescence, young adults, cancer, and psychological outcomes. All of the searches were reported in accordance with PRISMA-Search (Supplementary Table 2). Table 4 outlines the inclusion criteria regarding the study populations, interventions, comparators, outcomes, and settings. The population of interest was AYAs with cancer, including both patients and survivors. To accommodate the various definitions of AYAs, we included studies that defined participants as AYAs within a lower or upper age range of ±5 years from the National Cancer Institute definition (i.e., 15–39 years old)^6^. The outcomes were determined a priori based on four key domains of psychological functioning that are particularly pertinent among survivors of AYA-onset cancer: emotional health, social functioning, cancer-related cognitive impairment, and health behavior^5^.

**Table 4:** Study inclusion criteria

Population	Patients with cancer aged between 15 and 39 (±5 years) years at the time of diagnosis and time of intervention, male or female, undergoing or having completed active cancer treatment
Intervention	Digital health interventions (including mobile applications, text messaging, websites, wearable devices, video, videoconferences, chatrooms, etc.)
Comparator	Usual care, waitlist, or control intervention (i.e., internet access)
Outcomes	(1) Quality of life, anxiety, depressive symptoms, psychological distress, fatigue, self-perceived stress (2) Social support, self-efficacy (3) Cancer-related cognitive impairment (4) Physical activity, sleep
Setting	Randomized controlled trial

The following were excluded from the review: (1) study protocols, (2) studies lacking efficacy outcomes, (3) duplicate publications, (4) conference abstracts or dissertations, (5) studies focused on health outcome assessment or cancer screening, and (6) studies not published in English.

### Study selection process

All of the citations were transferred to EndNote software. After duplicates were removed, the titles and abstracts were independently reviewed by two reviewers based on the inclusion criteria. Abstracts with unclear information were included for full-text screening. Two reviewers examined the full texts of the articles and documented the reasons for exclusion. Disagreements were resolved through discussion until a consensus was reached, and if necessary, a third reviewer was consulted. If a single RCT reported outcomes of interest in multiple articles, all of the relevant articles were included.

### Data collection process

A standard data extraction sheet was developed in Word to record information from the included studies. Two authors (P.X. and Y.W.) independently extracted data on the study characteristics (author, year, country, study design), participant details (sample size, age, sex distribution, type of cancer, etc.), intervention specifics (content, duration, follow-up, etc.), type of comparator, outcomes (QoL, anxiety and depressive symptoms, psychological distress, self-efficacy, social support, fatigue, sleep, physical activity, and self-perceived stress), and Cochrane RoB measures.

### Risk of bias assessment

The RoB in the included articles was assessed using the Cochrane Collaboration RoB2 tool^81^. The domains evaluated were bias arising from the randomization process, bias due to deviations from intended interventions, bias due to missing outcome data, bias in the measurement of outcomes, and bias in the selection of the reported results. The criteria for categorizing RoB as low, having some concerns, or high followed the guidelines outlined in the Cochrane Handbook for Systematic Reviews of Interventions. The RoB was independently assessed by the authors (P.X. and Y.K.C.). Any disagreements were further discussed and resolved with a third reviewer (Y.T.C.) until a consensus was reached.

### Data synthesis

We summarized the characteristics of the included studies using descriptive tables. The means and standard deviations (SDs) of the primary and secondary outcomes at the end of the interventions for both the experimental and control groups were converted to SMDs using Hedges’ *G*. To account for the differences across studies due to the varied population and interventions, meta-analyses were conducted using random-effects models^82^. The restricted maximum likelihood method was used to estimate and pool the outcome SMDs (Hedges’ *G* method) and 95% CIs. For studies with missing or unclear data required to calculate effect estimates, the review team attempted to obtain clarification by contacting the primary study authors via email, with one reminder sent if no reply was received.

Quantitative heterogeneity was assessed using the between-study variance (*τ*^2^), the 95% prediction intervals (which provide an estimate of where true effects are expected for 95% of similar studies that may be conducted in the future), and *I*^2^ (the proportion of variability attributable to heterogeneity rather than sampling error). Additionally, we conducted a formal test of homogeneity based on Cochran’s *Q* test (with *p* < 0.1 considered statistically significant) and a series of univariate meta-regressions, treating the following variables as fixed-effect covariates: mean age, patient type (patients, patients and survivors, survivors), length of intervention (≤2 months, >2 months), condition of the control group (usual care, active/attention placebo control, waitlist), mode of delivery (self-guided, instructor-led), and RoB (low, some concerns, high). We also performed influence analyses using the leave-one-out method.

If studies included multiple intervention arms, only the most complex intervention (defined as having the largest number of intervention components^69^) was used in the meta-analyses. Some studies reported different measures for the same outcome. To address multiplicity, the reductionist approach was used to select the most commonly used measure across studies. Forest plots were generated for outcome variables that were included in three or more studies, while those included in two or fewer studies were summarized in the text. A funnel plot was used to detect publication bias when there were more than three studies per outcome, and the regression-based Egger’s test for funnel plot asymmetry was also conducted. Statistical analyses were performed using R Core Team (2020).

## Supplementary information

Supplementary information

## Data Availability

All data generated or analyzed during this study are included in this published article and its supplementary information files.
