# Supplementary material for: Digital health interventions for psychological outcomes among adolescents and young adults with cancer: a systematic review and meta-analysis
Source: NPJ Digit Med. 2026 May 9;9:727. doi: 10.1038/s41746-026-02719-x (PMC13612578; doi:10.1038/s41746-026-02719-x)
Supplement: Supplementary file 1 [file 41746_2026_2719_MOESM1_ESM.pdf]

Digital health interventions for psychological outcomes among adolescents and young adults with cancer: a systematic review and meta-analysis

Panpan Xiao, Yihui Wei, Yan Kate Chow, Keary Rui Zhou, Winnie Wan-Yee Tso, Kevin Yi-Lwern Yap, Herbert Ho Fung Loong, Chi Kong Li, Yin Ting Cheung

Table of Contents

Section S1: Supplementary Figures

Supplementary Figure 1: Sensitivity analysis for quality of life
Supplementary Figure 2: Sensitivity analysis for anxiety symptoms
Supplementary Figure 3: Sensitivity analysis for depressive symptoms
Supplementary Figure 4: Sensitivity analysis for psychological distress
Supplementary Figure 5: Sensitivity analysis for self-efficacy
Supplementary Figure 6: Sensitivity analysis for social support
Supplementary Figure 7: Sensitivity analysis for fatigue
Supplementary Figure 8: Funnel plot for quality of life
Supplementary Figure 9: Funnel plot for anxiety symptoms
Supplementary Figure 10: Funnel plot for depressive symptoms
Supplementary Figure 11: Funnel plot for psychological distress
Supplementary Figure 12: Funnel plot for self-efficacy
Supplementary Figure 13: Funnel plot for social support
Supplementary Figure 14: Funnel plot for fatigue

Section S2: Supplementary Tables

Supplementary Table 1: Univariable meta-regression of moderators for psychological outcomes in adolescents and young adults with cancer
Supplementary Table 2: Search strategies for six databases
Supplementary Table 3: PRISMA 2020 checklist

Section S1: Supplementary Figure

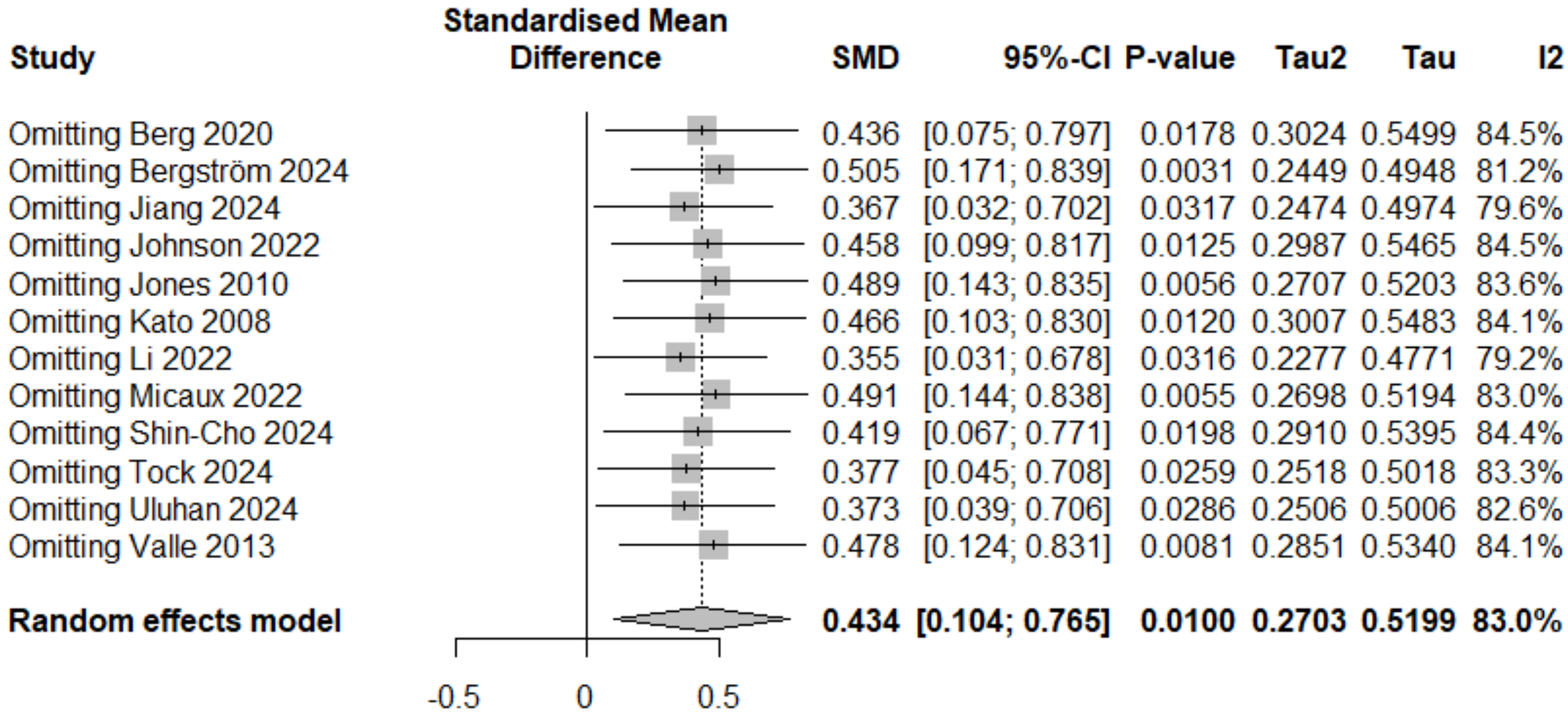

Supplementary Figure 1. Sensitivity analysis for quality of life. Each row represents the pooled effect size (standardised mean difference, SMD) and heterogeneity estimates after omitting one study at a time. The results demonstrated that the pooled estimates remain relatively robust to the exclusion of individual studies.

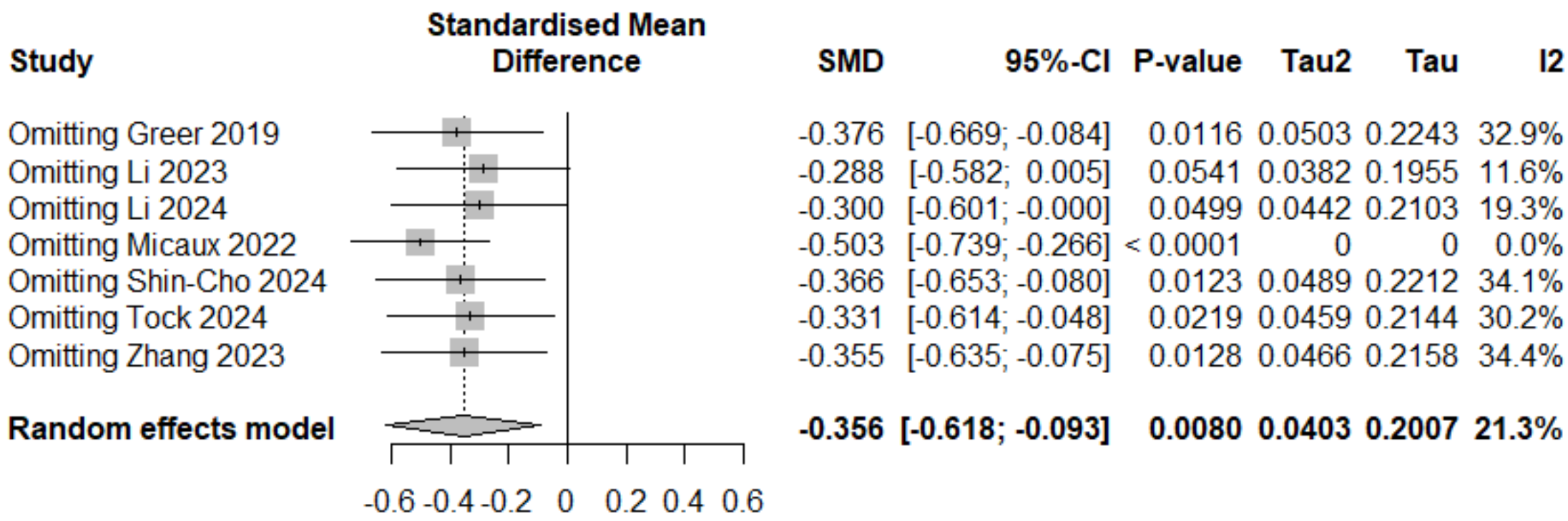

Supplementary Figure 2. Sensitivity analysis for anxiety symptoms. The results demonstrated that the pooled estimates remained relatively robust to the exclusion of individual studies.

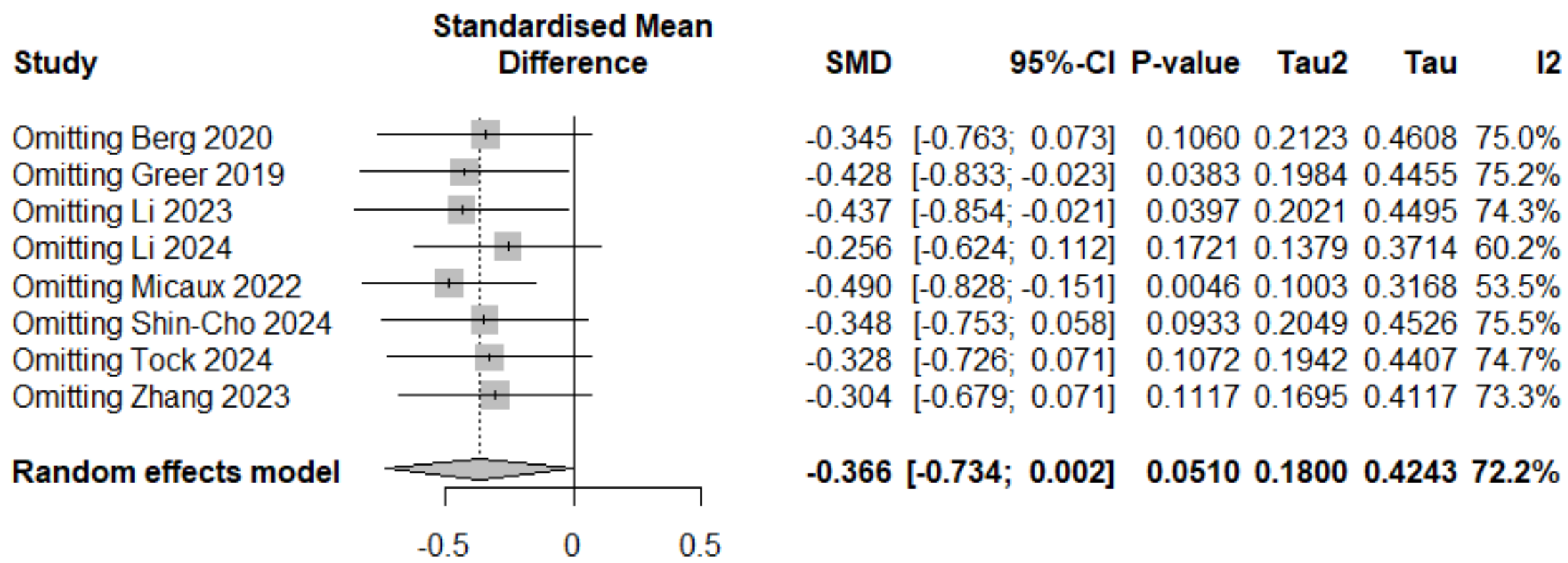

Supplementary Figure 3. Sensitivity analysis for depressive symptoms. The results

revealed that the pooled effect estimates were not fully robust and may be influenced by specific studies, indicating potential instability in the overall findings.

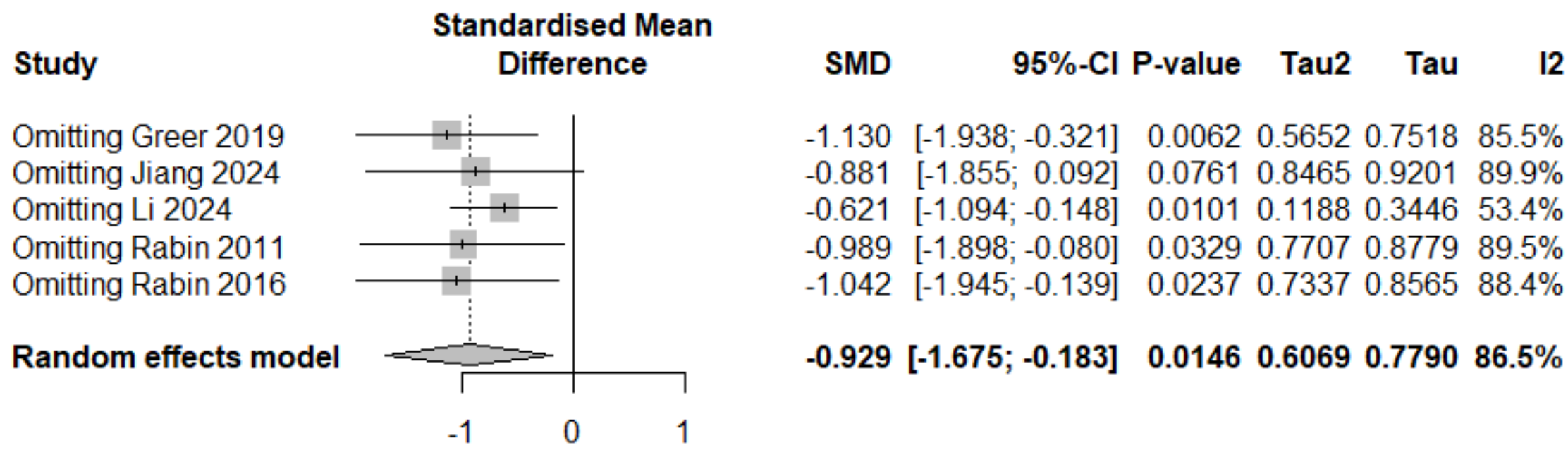

Supplementary Figure 4. Sensitivity analysis for psychological distress. The results revealed that the pooled effect estimates were not fully robust and may be influenced by Jiang et al’s study, indicating potential instability in the overall findings.

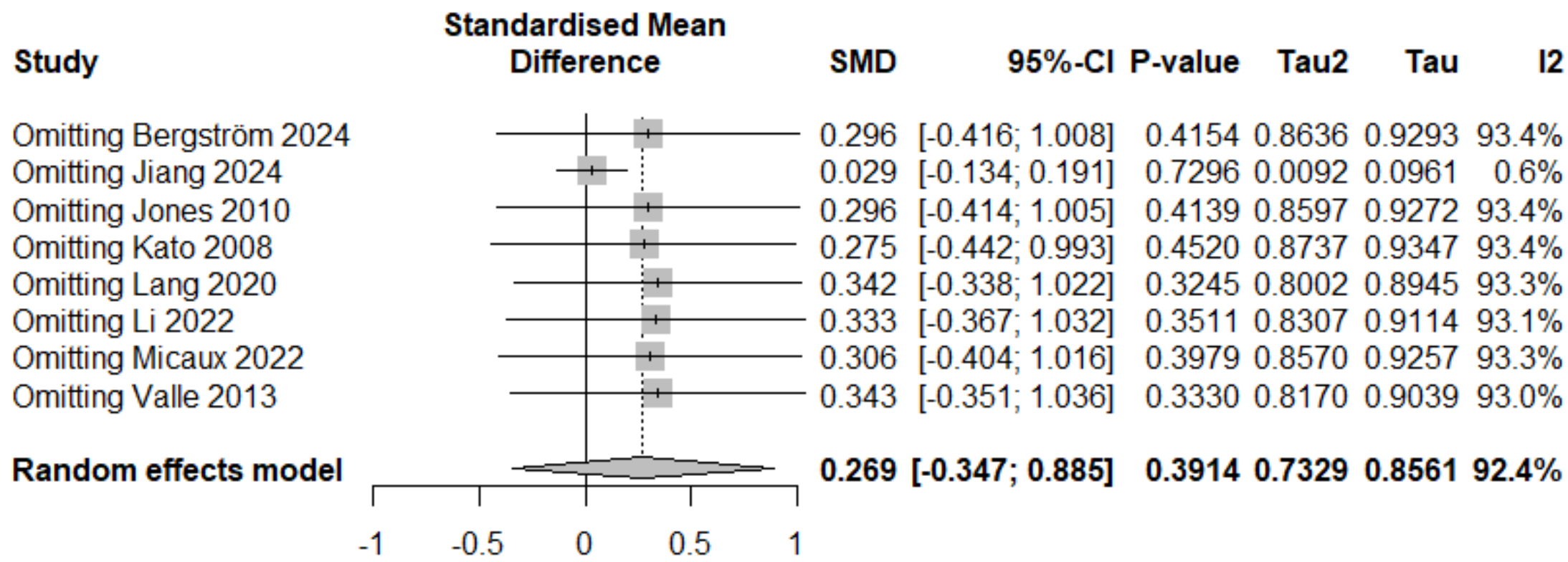

Supplementary Figure 5. Sensitivity analysis for self-efficacy. The results demonstrated that the pooled estimates remained relatively robust to the exclusion of individual studies.

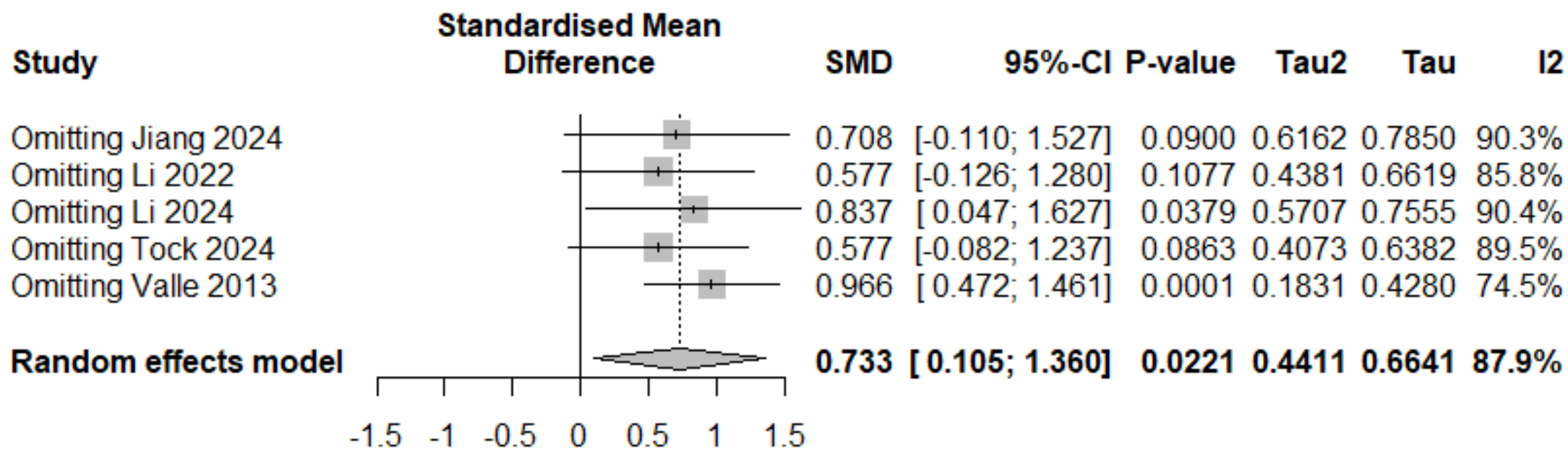

Supplementary Figure 6. Sensitivity analysis for social support. The results revealed that the pooled effect estimates were not fully robust and may be influenced by Valle et al’s study, indicating potential instability in the overall findings.

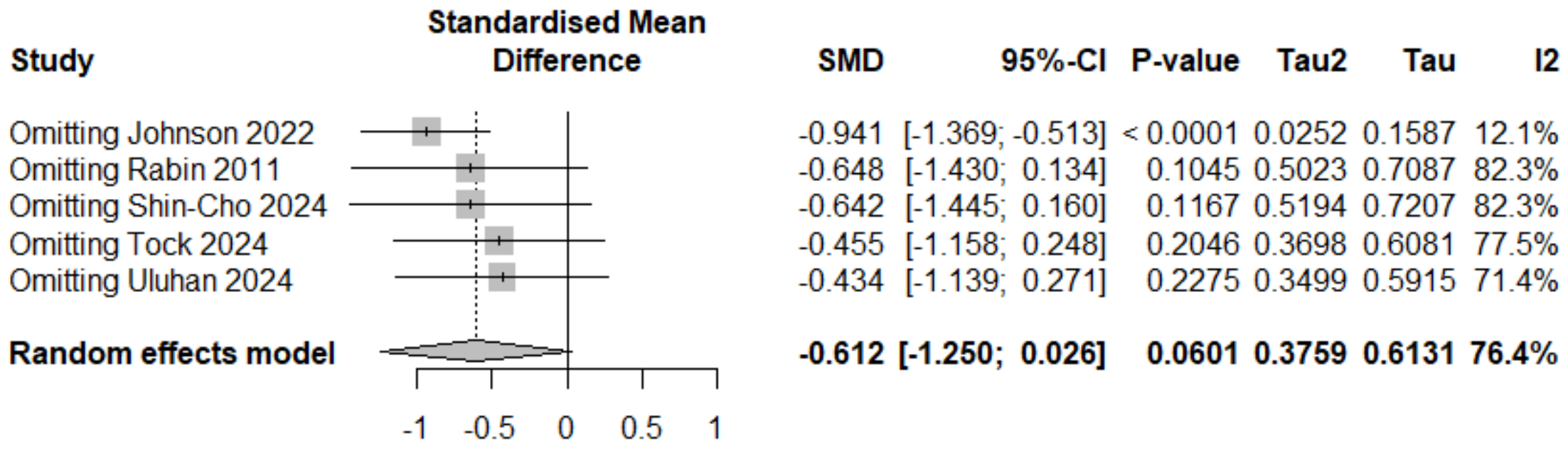

Supplementary Figure 7. Sensitivity analysis for Fatigue. The results revealed that the pooled effect estimates were not fully robust and may be influenced by Johnson et al's study, indicating potential instability in the overall findings.

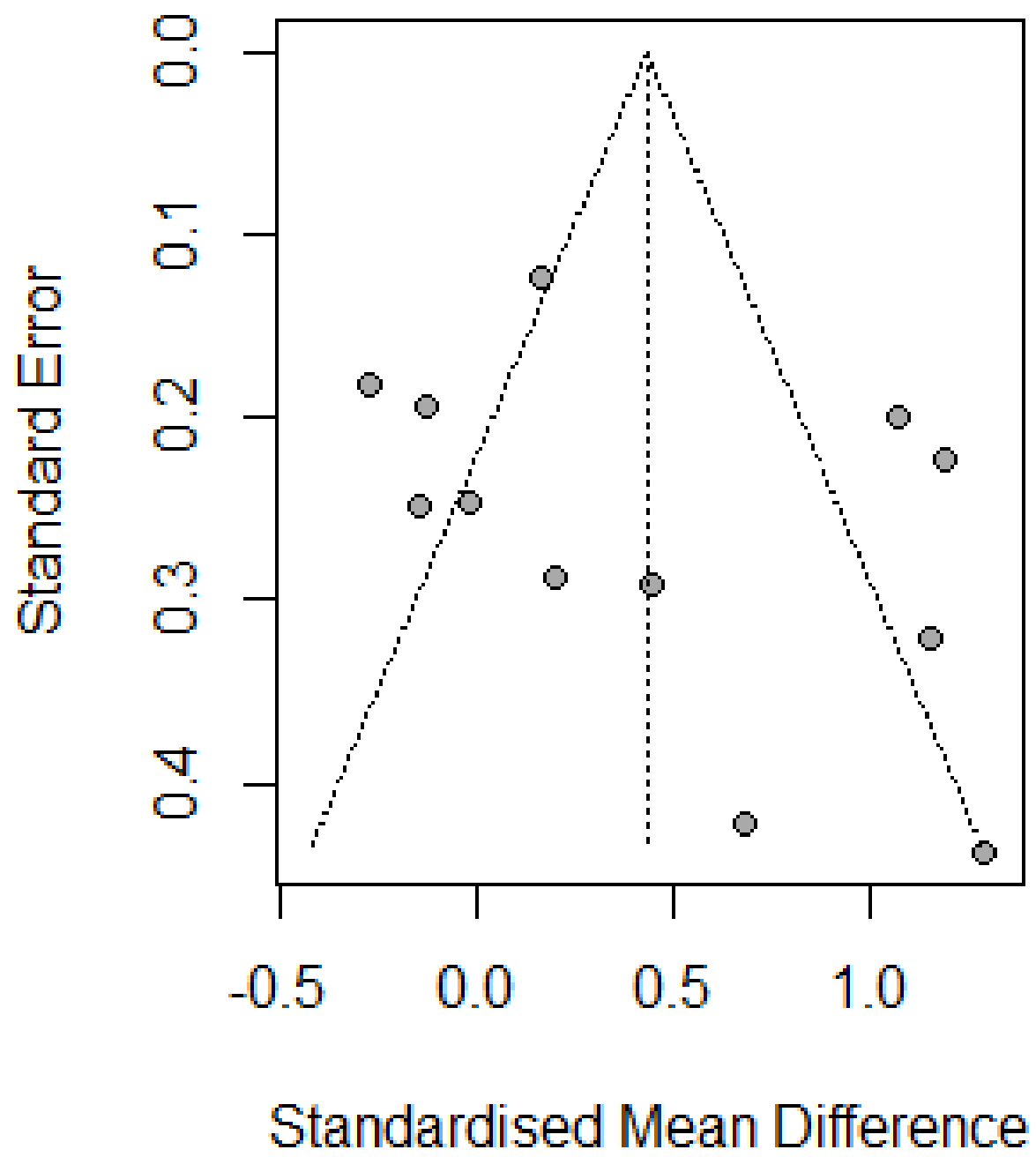

Supplementary Figure 8. Funnel plot for quality of life. Each circle represents an individual study, with the x-axis depicting the standardised mean difference (SMD) and the y-axis displaying the standard error (SE) of the SMD. The vertical dashed line indicates the overall effect size. The plot shows a relatively symmetrical distribution of studies around the central effect estimate. This is further supported by Egger's regression test ($t = 1.31$, $df = 10$, $p = 0.22$).

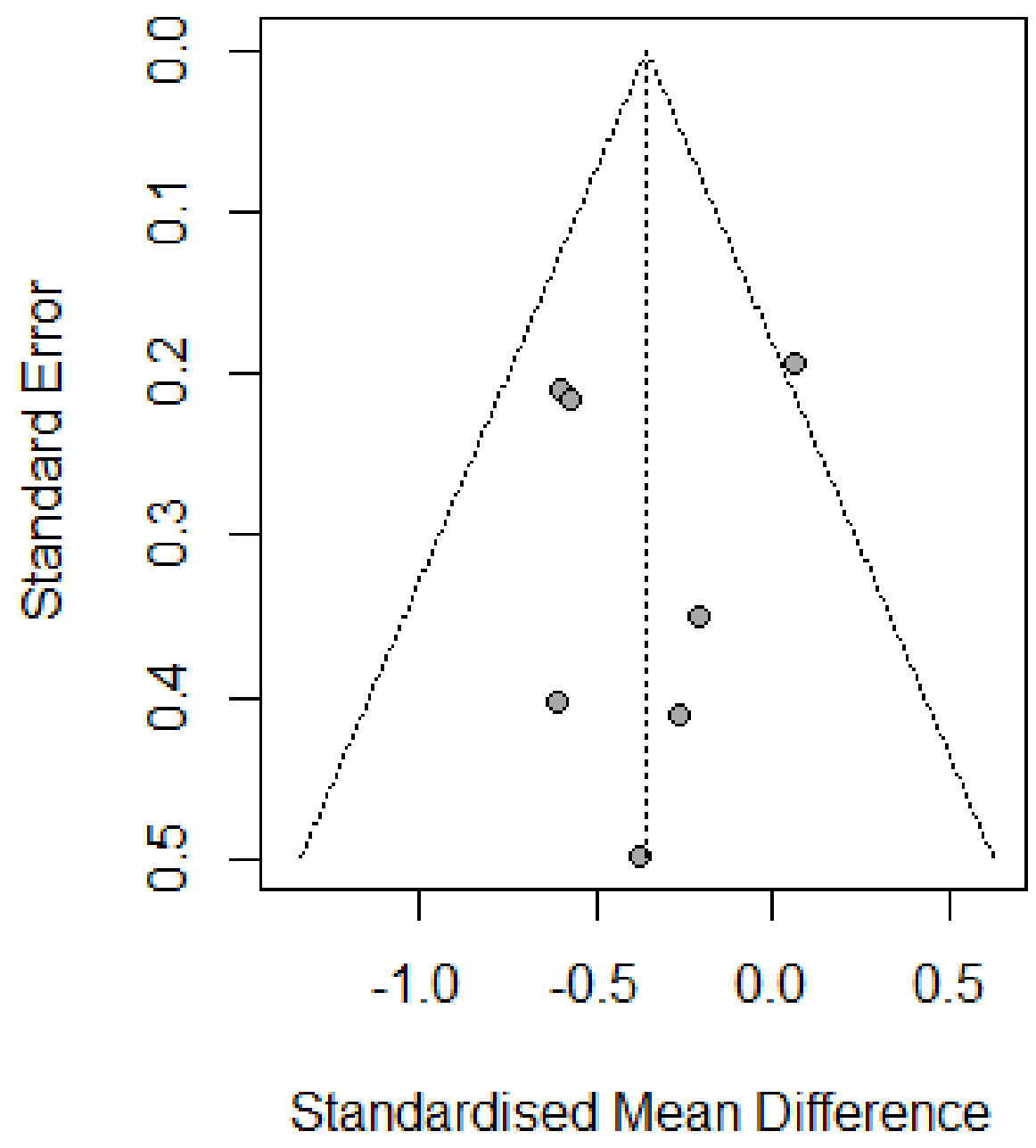

Supplementary Figure 9. Funnel plot for anxiety symptoms. The Egger's regression test did not indicate significant asymmetry (t= -0.28, df = 5, p = 0.79).

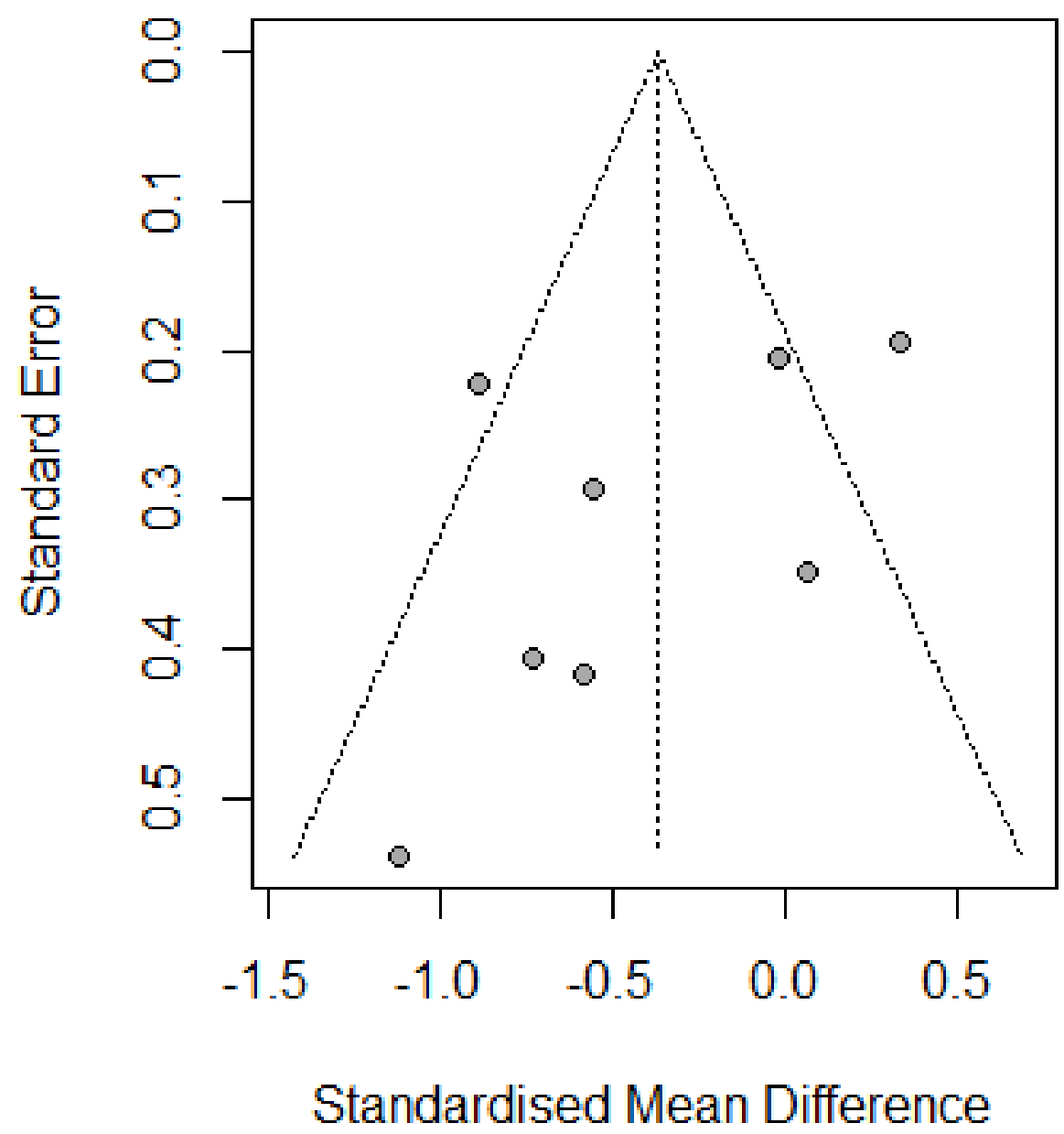

Supplementary Figure 10. Funnel plot for anxiety symptoms. The Egger's regression test did not indicate significant asymmetry (t= -1.40, df = 6, p = 0.21).

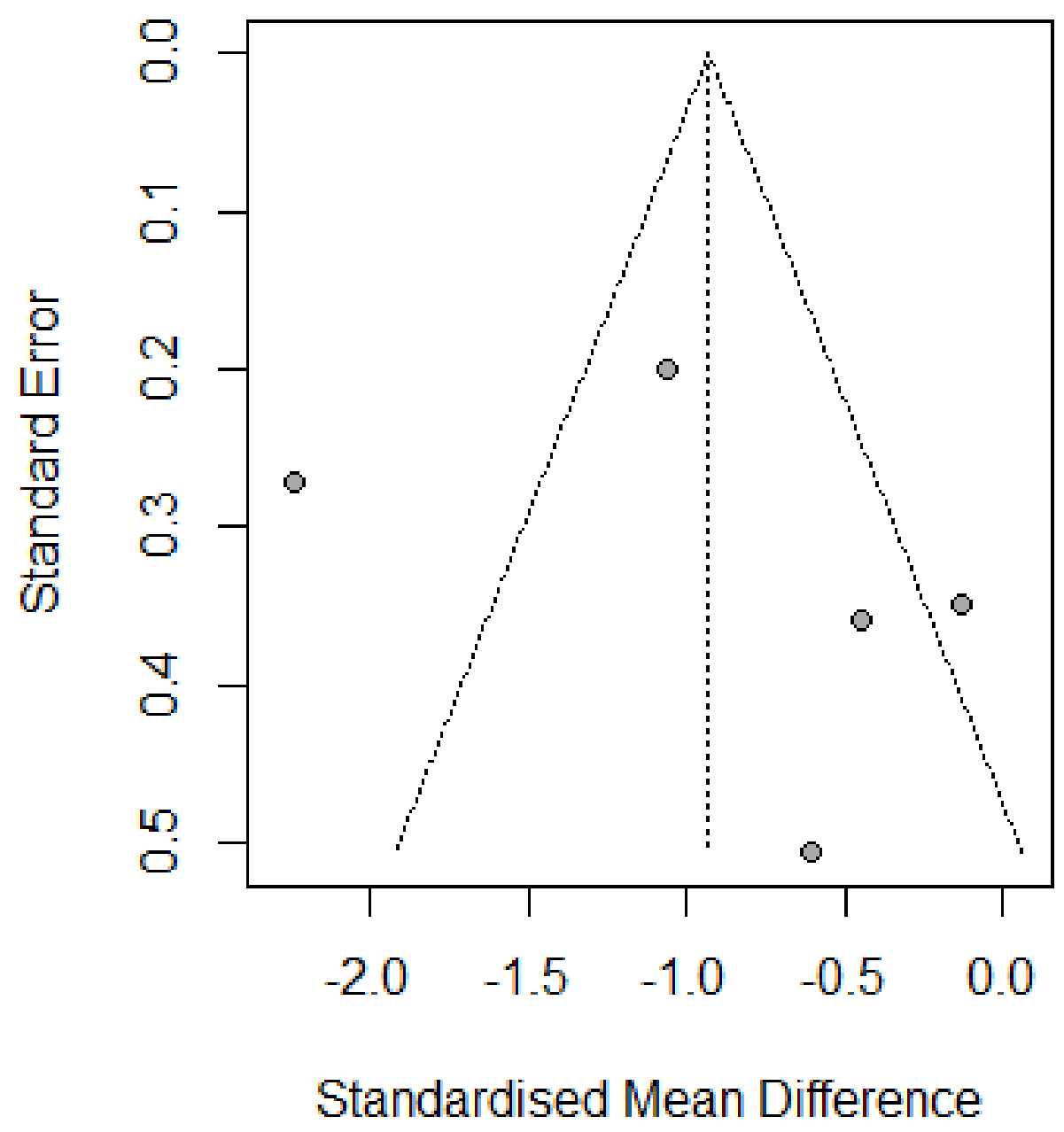

Supplementary Figure 11. Funnel plot for psychological distress. The Egger's regression test did not indicate significant asymmetry ($t = 0.75$, $df = 3$, $p = 0.51$)

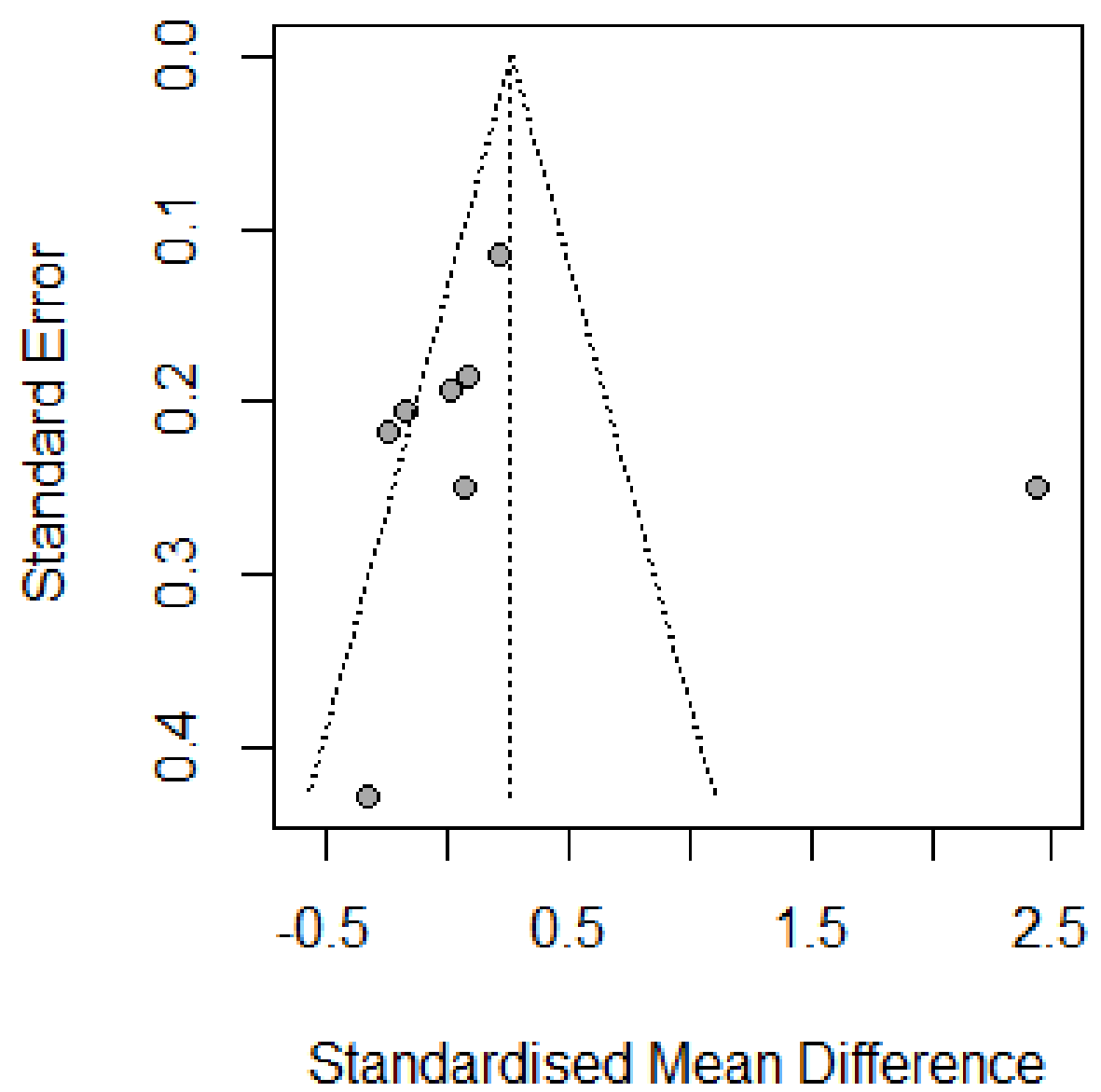

Supplementary Figure 12. Funnel plot for self-efficacy. The Egger's regression test did not indicate significant asymmetry ($t = 0.24$, $df = 6$, $p = 0.82$).

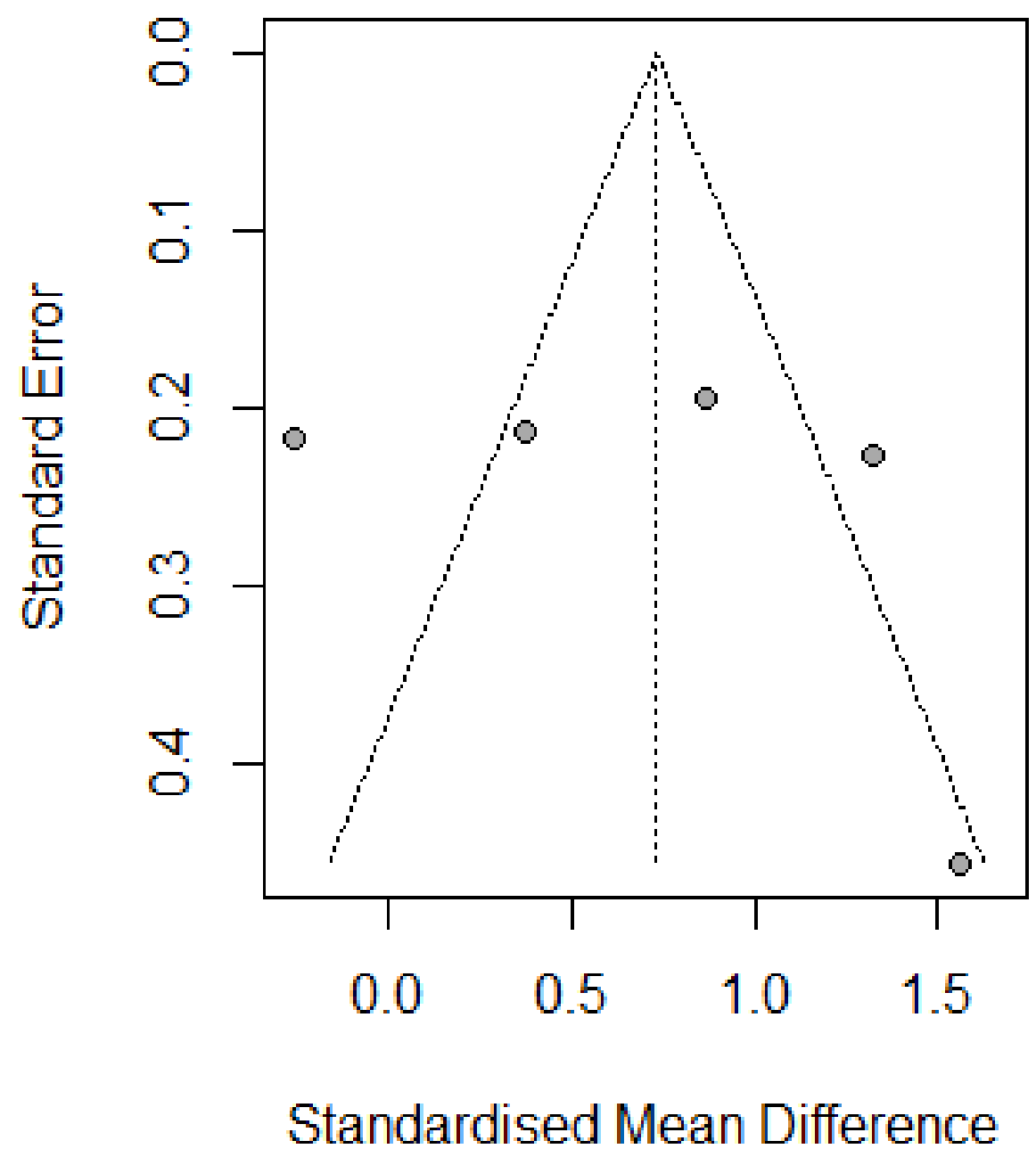

Supplementary Figure 13. Funnel plot for self-efficacy. The Egger's regression test did not indicate significant asymmetry (t = 0.67, df = 3, p = 0.55)

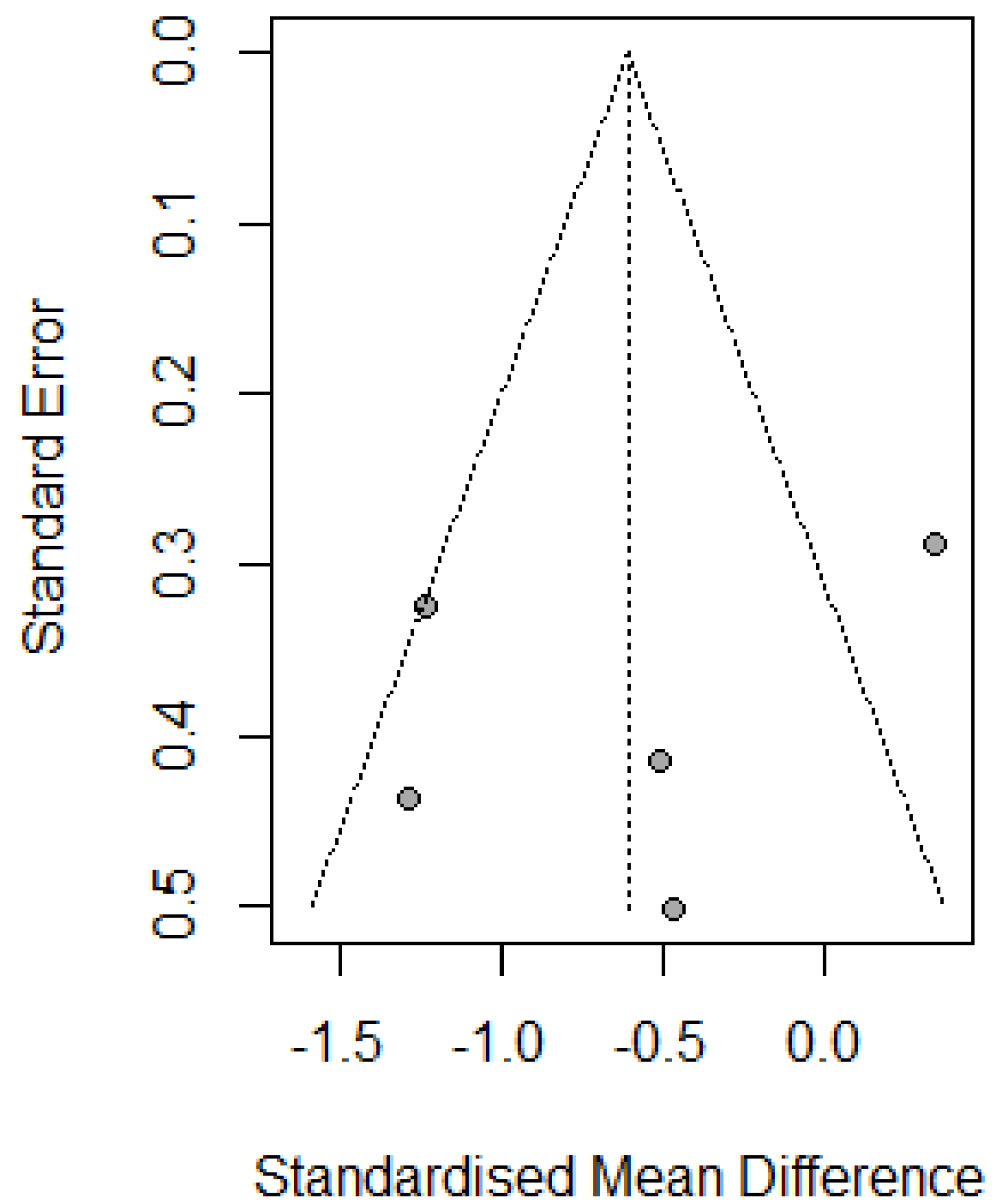

Supplementary Figure 14. Funnel plot for self-efficacy. The Egger's regression test did not indicate significant asymmetry (t = -0.80, df = 3, p = 0.48)

Section S2: Supplementary Tables

Supplementary Table 1. Univariable meta-regression of moderators for psychological outcomes in adolescents and young adults with cancer

Outcomes	Moderators	Levels	Estimate (95% CI)	τ^2	I^2	Test for residual heterogeneity p-value	Test of moderators p-value
**Quality of life**							
	Mean age		0.007 (-0.034, 0.047)	0.300	83.99%	<0.0001	0.756
	Patient type	Patients & Survivors *vs*. Patients	-0.278 (-1.300, 0.745)	0.331	84.27%	<0.0001	0.854
		Survivors *vs*. Patients	-0.129 (-1.158, 0.899)				
	Condition of control group	Usual care *vs*. Attention placebo control	0.037 (-0.657, 0.732)	0.305	84.75%	<0.0001	0.946
	Intervention period	≤2 months *vs*. >2 months	0.478 (-0.275, 1.231)	0.244	82.59%	<0.0001	0.213
	Mode of delivery	Self-guided *vs*. Instructor-led	-0.506 (-1.150, 0.137)	0.231	81.77%	<0.0001	0.123
	Risk of bias	Some concerns *vs*. Low	0.334 (-0.366, 1.03)	0.268	83.73%	<0.0001	0.350
**Anxiety symptoms**							
	Mean age		0.016 (-0.057, 0.089)	0.042	35.61%	0.233	0.665
	Patient type	Survivors vs. Patients & Survivors	0.160 (-0.522, 0.842)	0.050	40.05%	0.192	0.646
	Condition of control group	Usual care vs. Active/attention placebo control	-0.084 (-0.876, 0.708)	0.063	48.51%	0.115	0.835
	Intervention period	≤2 months vs. >2 months	-0.426 (-0.848, -0.005)	0	0	0.593	**0.048**
	Mode of delivery	Self-guided vs. Instructor-led	0.541 (0.124, 0.957)	0	0	0.950	**0.011**
	Risk of bias	Some concerns vs. Low	-0.152 (-0.704, 0.399)	0.045	34.08%	0.254	0.589
**Depressive symptoms**							
	Mean age		0.024 (-0.074, 0.122)	0.204	75.50%	0.0005	0.637
	Patient type	Survivors vs. Patients & Survivors	0.030 (-0.796, 0.856)	0.221	73.35%	0.0004	0.943

	Condition of control group	Usual care vs. Active/attention placebo control	0.425 (-0.422, 1.273)	0.198	72.46%	0.001	0.325
	Intervention period	≤2 months vs. >2 months	-0.395 (-1.214, 0.424)	0.170	65.22%	0.006	0.345
	Mode of delivery	Self-guided vs. Instructor-led	0.802 (0.180, 1.424)	0.074	45.15%	0.095	**0.012**
	Risk of bias	Some concerns vs. Low	0.100 (-0.686, 0.886)	0.218	71.40%	0.0003	0.803
**Psychological distress**							
	Mean age		-0.027 (-0.195, 0.141)	0.821	87.44%	<0.0001	0.753
	Condition of control group	Waitlist vs. Usual care	1.339 (0.061, 2.616)	0.334	81.09%	0.002	**0.040**
	Intervention period	≤2 months vs. >2 months	-0.489 (-2.140, 1.161)	0.740	87.28%	<0.0001	0.561
	Mode of delivery	Self-guided vs. Instructor-led	0.757 (-0.762, 2.275)	0.609	85.02%	<0.0001	0.329
	Risk of bias	Some concerns vs. Low	1.339 (0.061, 2.616)	0.334	81.09%	0.002	**0.040**
**Self-efficacy**							
	Mean age		0.041 (-0.032, 0.113)	0.714	93.89%	<0.0001	0.272
	Patient type	Survivors vs. Patients & Survivors	1.142 (-0.282, 2.566)	0.696	93.02%	<0.0001	0.116
	Condition of control group	Usual care vs. Attention placebo control	-0.788 (-2.131, 0.555)	0.763	95.09%	<0.0001	0.250
	Mode of delivery	Self-guided vs. Instructor-led	0.666 (-0.812, 2.144)	0.762	94.98%	<0.0001	0.377
	Risk of bias	Some concerns vs. Low	-0.420 (-1.917, 1.077)	0.821	95.27%	<0.0001	0.583
**Social support**							
	Mean age		0.012 (-0.174, 0.199)	0.624	90.83%	<0.0001	0.896
	Patient type	Survivors vs. Patients & Survivors	-0.727 (-1.972, 0.517)	0.422	87.66%	<0.0001	0.252
	Condition of control group	Usual care vs. Attention placebo control	0.727 (-0.517, 1.972)	0.422	87.66%	<0.0001	0.252

	Intervention period	≤2 months vs. >2 months	0.179 (-1.281, 1.639)	0.600	91.02%	<0.0001	0.810
**Fatigue**	Mode of delivery	Self-guided vs. Instructor-led	-0.727 (-1.972, 0.517)	0.422	87.66%	<0.0001	0.252
	Mean age		0.045 (-0.032, 0.122)	0.327	67.10%	0.019	0.252
	Condition of control group	Usual care vs. Attention placebo control	-1.167 (-1.972, -0.361)	0.060	28.28%	0.284	**0.005**
	Mode of delivery	Self-guided vs. Instructor-led	-0.464 (-1.826, 0.898)	0.420	73.42%	0.008	0.505
	Risk of bias	Some concerns vs. Low	-0.464 (-1.826, 0.898)	0.420	73.42%	0.008	0.505

Supplementary Table 2. Search strategies for six databases

Item	Search strategies	Results
**PubMed (1976-, Search on 4 January 2025)**		
#1	(((Neoplasms[MeSH Terms]) OR (Carcinoma[MeSH Terms])) OR (Cancer survivors[MeSH Terms])) OR (cancer*[Title/Abstract] OR oncolog*[Title/Abstract] OR neoplasm*[Title/Abstract] OR carcinoma*[Title/Abstract] OR tumo*[Title/Abstract] OR malignanc*[Title/Abstract] OR melanoma[Title/Abstract] OR sarcoma[Title/Abstract] OR adenocarcinoma*[Title/Abstract] OR glioma*[Title/Abstract] OR lymphoma*[Title/Abstract] OR myeloma*[Title/Abstract] OR leukemia*[Title/Abstract] OR leucaemia*[Title/Abstract])	5,500,569
#2	((Adolescent[MeSH Terms]) OR (Young Adult[MeSH Terms])) OR (adolescen*[Title/Abstract] OR youth*[Title/Abstract] OR teen*[Title/Abstract] OR teenager*[Title/Abstract] OR young adult*[Title/Abstract] OR AYA[Title/Abstract])	2,986,204
#3	((((((((((((((((((Digital Health[MeSH Terms]) OR (Telemedicine[MeSH Terms])) OR (Telecommunications[MeSH Terms])) OR (Electronic Mail[MeSH Terms])) OR (Videoconferencing[MeSH Terms])) OR (Cell Phone[MeSH Terms])) OR (Smartphone[MeSH Terms])) OR (Text Messaging[MeSH Terms])) OR (Computers[MeSH Terms])) OR (Internet[MeSH Terms])) OR (Social Media[MeSH Terms])) OR (Mobile Applications[MeSH Terms])) OR (Video Games[MeSH Terms])) OR (Multimedia[MeSH Terms])) OR (Video Recording[MeSH Terms])) OR (CD-ROM[MeSH Terms])) OR (Virtual Reality[MeSH Terms])) OR (Fitness Trackers[MeSH Terms])) OR (Wearable Electronic Devices[MeSH Terms])) OR (Internet-Based Intervention[MeSH Terms])	383,126
#4	digital health[Title/Abstract] OR telemedicine[Title/Abstract] OR tele-medicine[Title/Abstract] OR telecare[Title/Abstract] OR tele-care[Title/Abstract] OR mobile health[Title/Abstract] OR mhealth[Title/Abstract] OR m-health[Title/Abstract] OR telehealth[Title/Abstract] OR tele-health[Title/Abstract] OR ehealth[Title/Abstract] OR electronic health*[Title/Abstract] OR e-health[Title/Abstract] OR virtual[Title/Abstract] OR online[Title/Abstract] OR telehomecare[Title/Abstract] OR tele-homecare[Title/Abstract] OR telehealthcare[Title/Abstract] OR tele-healthcare[Title/Abstract] OR telemonitoring[Title/Abstract] OR tele-monitoring[Title/Abstract] OR telecommunication*[Title/Abstract] OR teleconference[Title/Abstract] OR podcast*[Title/Abstract] OR webcast*[Title/Abstract] OR email*[Title/Abstract] OR e-mail[Title/Abstract] OR videoconferenc*[Title/Abstract] OR video conference*[Title/Abstract] OR cell phone[Title/Abstract] OR telephone[Title/Abstract] OR mobile phone[Title/Abstract] OR smartphone[Title/Abstract] OR smart phone[Title/Abstract] OR texting*[Title/Abstract] OR text messag*[Title/Abstract] OR short messag*[Title/Abstract] OR SMS[Title/Abstract] OR computer*[Title/Abstract] OR internet[Title/Abstract] OR world wide web[Title/Abstract] OR website*[Title/Abstract] OR web browser[Title/Abstract] OR internet-based intervention*[Title/Abstract] OR internet intervention*[Title/Abstract] OR web-based intervention*[Title/Abstract] OR social media[Title/Abstract] OR Twitter[Title/Abstract] OR Facebook[Title/Abstract] OR Instagram[Title/Abstract] OR software*[Title/Abstract] OR computer program*[Title/Abstract] OR computer software[Title/Abstract] OR computer application[Title/Abstract] OR mobile application*[Title/Abstract] OR mobile App[Title/Abstract] OR portable electronic App[Title/Abstract] OR smartphone App[Title/Abstract] OR software application*[Title/Abstract] OR electronic	1,311,886

	application*[Title/Abstract] OR software App[Title/Abstract] OR video game*[Title/Abstract] OR computer game*[Title/Abstract] OR video recording[Title/Abstract] OR videorecording*[Title/Abstract] OR iPhone[Title/Abstract] OR Android[Title/Abstract] OR iPad[Title/Abstract] OR CD-ROM[Title/Abstract] OR e-learning[Title/Abstract] OR elearning[Title/Abstract] OR chatbot[Title/Abstract] OR online chat*[Title/Abstract] OR wearable device*[Title/Abstract] OR wearable electronic device*[Title/Abstract] OR wearable technolog*[Title/Abstract] OR fitness tracker*[Title/Abstract] OR activity tracker*[Title/Abstract]	
#5	#3 OR #4	1,493,028
#6	#1 AND #2 AND #5	13,482
#7	Filters applied: Clinical Trial, Clinical Trial, Phase I, Clinical Trial, Phase II, Clinical Trial, Phase III, Clinical Trial, Phase IV, Controlled Clinical Trial, Randomized Controlled Trial.	1,015
**Embase (OVID) (1910-, Search on 4 January 2025)**		
#1	exp neoplasm/ or exp carcinoma/ or exp malignant neoplasm/ or (cancer* or oncolog* or neoplasm* or carcinoma* or tumo* or melanoma or sarcoma or adenocarcinoma* or glioma* or lymphoma* or myeloma* or leukemia* or leucaemia*).ti,ab.	7280816
#2	exp adolescent/ or exp adolescence/ or exp young adult/ or (adolescen* or youth* or teen* or teenager* or young adult* or AYA).ti,ab.	2553008
#3	exp digital health/ or exp telehealth/ or exp telemedicine/ or exp telecommunication/ or exp mobile application/ or exp internet/ or exp computer/ or exp web-based intervention/ or exp telephone/ or exp mobile phone/ or exp text messaging/ or exp video game/ or exp computer program/ or exp videoconferencing/ or exp podcast/ or exp CD-ROM/ or exp social media/ or exp multimedia/ or exp videorecording/ or exp virtual reality/ or exp wearable device/ or exp activity tracker/ or (digital health or telemedicine or tele-medicine or telecare or tele-care or mobile health or mhealth or m-health or telehealth or tele-health or ehealth or electronic health* or e-health or virtual or online or telehomecare or tele-homecare or telehealthcare or tele-healthcare or telemonitoring or tele-monitoring or telecommunication* or teleconference or podcast* or webcast* or email* or e-mail or videoconferenc* or video conference* or cell phone or telephone or mobile phone or smartphone or smart phone or texting* or text messag* or short messag* or SMS or computer* or internet or world wide web or website* or web browser or internet-based intervention* or internet intervention* or web-based intervention* or social media or Twitter or Facebook or Instagram or software* or computer program* or computer software or computer application or mobile application* or mobile App or portable electronic App or smartphone App or software application* or electronic application* or software App or video game* or computer game* or video recording or videorecording* or iPhone or Android or iPad or CD-ROM or e-learning or elearning or chatbot or online chat* or wearable device* or wearable electronic device* or wearable technolog* or fitness tracker* or activity tracker*).ti,ab.	2361854
#4	#1 and #2 and #3	19670
#5	limit #4 to (clinical trial or randomized controlled trial or controlled clinical trial or multicenter study or phase 1 clinical trial or phase 2 clinical trial or phase 3 clinical trial or phase 4 clinical trial)	1760
**Cochrane Library (1996-, Search on 4 January 2025)**		
#1	MeSH descriptor: [Neoplasms] explode all trees OR MeSH descriptor: [Carcinoma] explode all trees OR (cancer* OR oncolog* OR	310791

	neoplasm* OR carcinoma* OR tumo* OR melanoma OR sarcoma OR adenocarcinoma* OR glioma* OR lymphoma* OR myeloma* OR leukemia* OR leucaemia*):ti,ab,kw	
#2	MeSH descriptor: [Adolescent] explode all trees OR MeSH descriptor: [Young Adult] explode all trees OR (adolescen* OR youth* OR teen* OR teenager* OR young adult* OR AYA):ti,ab,kw	262090
#3	MeSH descriptor: [Digital Health] explode all trees OR MeSH descriptor: [Telemedicine] explode all trees OR MeSH descriptor: [Telecommunications] explode all trees OR MeSH descriptor: [Electronic Mail] explode all trees OR MeSH descriptor: [Videoconferencing] explode all trees OR MeSH descriptor: [Cell Phone] explode all trees OR MeSH descriptor: [Smartphone] explode all trees OR MeSH descriptor: [Text Messaging] explode all trees OR MeSH descriptor: [Computers] explode all trees OR MeSH descriptor: [Internet] explode all trees OR MeSH descriptor: [Internet-Based Intervention] explode all trees OR MeSH descriptor: [Social Media] explode all trees OR MeSH descriptor: [Multimedia] explode all trees OR MeSH descriptor: [Mobile Applications] explode all trees OR MeSH descriptor: [Video Games] explode all trees OR MeSH descriptor: [Video Recording] explode all trees OR MeSH descriptor: [CD-ROM] explode all trees OR MeSH descriptor: [Virtual Reality] explode all trees OR MeSH descriptor: [Wearable Electronic Devices] explode all trees MeSH descriptor: [Fitness Trackers] explode all trees	346927
#4	(digital health OR telemedicine OR tele-medicine OR telecare OR tele-care OR mobile health OR mhealth OR m-health OR telehealth OR tele-health OR ehealth OR electronic health* OR e-health OR virtual OR online OR telehomecare OR tele-homecare OR telehealthcare OR tele-healthcare OR telemonitoring OR tele-monitoring OR telecommunication* OR teleconference OR podcast* OR webcast* OR email* OR e-mail OR videoconferenc* OR video conference* OR cell phone OR telephone OR mobile phone OR smartphone OR smart phone OR texting* OR text messag* OR short messag* OR SMS OR computer* OR internet OR world wide web OR website* OR web browser OR internet-based intervention* OR internet intervention* OR web-based intervention* OR social media OR Twitter OR Facebook OR Instagram OR software* OR computer program* OR computer software OR computer application OR mobile application* OR mobile App OR portable electronic App OR smartphone App OR software application* OR electronic application* OR software App OR video game* OR computer game* OR video recording OR videorecording* OR iPhone OR Android OR iPad OR CD-ROM OR e-learning OR elearning OR chatbot OR online chat* OR wearable device* OR wearable electronic device* OR wearable technolog* OR fitness tracker* OR activity tracker*):ti,ab,kw	220891
#5	#3 OR #4	511677
#6	#1 AND #2 AND #5	6754
#7	(randomized OR randomly OR randomization OR controlled clinical trial* OR controlled trial* OR clinical trial* OR multicenter trial*):ti,ab,kw	1505852
#8	#6 AND #7	5831
**CINAHL (EBSCO) (1937-, Search on 4 January 2025)**		
S1	(MH "Neoplasms+") OR (MH "Carcinoma") OR TI (cancer* OR oncolog* OR neoplasm* OR carcinoma* OR tumo* OR melanoma OR sarcoma OR adenocarcinoma* OR glioma* OR lymphoma* OR myeloma* OR leukemia* OR leucaemia*) OR AB (cancer* OR oncolog* OR neoplasm* OR carcinoma* OR tumo* OR melanoma OR sarcoma OR adenocarcinoma* OR glioma* OR lymphoma*	315,755

	OR myeloma* OR leukemia* OR leucaemia*)	
S2	(MH "Young Adult") OR (MH "Adolescence") OR TI (adolescen* OR youth* OR teen* OR teenager* OR young adult* OR AYA) OR AB (adolescen* OR youth* OR teen* OR teenager* OR young adult* OR AYA)	850,561
S3	(MH "Digital Health+") OR (MH "Telemedicine") OR (MH "Telehealth") OR (MH "Telecommunications") OR (MH "Teleconferencing") OR (MH "Email") OR (MH "Videoconferencing") OR (MH "Cellular Phone") OR (MH "Telephone") OR (MH "Mobile Applications") OR (MH "Text Messaging") OR (MH "Smartphone") OR (MH "Internet") OR (MH "World Wide Web") OR (MH "World Wide Web Applications") OR (MH "Internet-Based Intervention") OR (MH "Social Media") OR (MH "Video Games") OR (MH "Videorecording") OR (MH "Video-Assisted Techniques and Procedures") OR (MH "Multimedia") OR (MH "CD ROM") OR (MH "Electrical Equipment and Supplies") OR (MH "Electrical Equipment and Supplies") OR (MH "Fitness Trackers")	284,825
S4	TI (digital health OR telemedicine OR tele-medicine OR telecare OR tele-care OR mobile health OR mhealth OR m-health OR telehealth OR tele-health OR ehealth OR electronic health* OR e-health OR virtual OR online OR telehomecare OR tele-homecare OR telehealthcare OR tele-healthcare OR telemonitoring OR tele-monitoring OR telecommunication* OR teleconference OR podcast* OR webcast* OR email* OR e-mail OR videoconferenc* OR video conference* OR cell phone OR telephone OR mobile phone OR smartphone OR smart phone OR texting* OR text messag* OR short messag* OR SMS OR computer* OR internet OR world wide web OR website* OR web browser OR internet-based intervention* OR internet intervention* OR web-based intervention* OR social media OR Twitter OR Facebook OR Instagram OR software* OR computer program* OR computer software OR computer application OR mobile application* OR mobile App OR portable electronic App OR smartphone App OR software application* OR electronic application* OR software App OR video game* OR computer game* OR video recording OR videorecording* OR iPhone OR Android OR iPad OR CD-ROM OR e-learning OR elearning OR chatbot OR online chat* OR wearable device* OR wearable electronic device* OR wearable technolog* OR fitness tracker* OR activity tracker*) OR AB (digital health OR telemedicine OR tele-medicine OR telecare OR tele-care OR mobile health OR mhealth OR m-health OR telehealth OR tele-health OR ehealth OR electronic health* OR e-health OR virtual OR online OR telehomecare OR tele-homecare OR telehealthcare OR tele-healthcare OR telemonitoring OR tele-monitoring OR telecommunication* OR teleconference OR podcast* OR webcast* OR email* OR e-mail OR videoconferenc* OR video conference* OR cell phone OR telephone OR mobile phone OR smartphone OR smart phone OR texting* OR text messag* OR short messag* OR SMS OR computer* OR internet OR world wide web OR website* OR web browser OR internet-based intervention* OR internet intervention* OR web-based intervention* OR social media OR Twitter OR Facebook OR Instagram OR software* OR computer program* OR computer software OR computer application OR mobile application* OR mobile App OR portable electronic App OR smartphone App OR software application* OR electronic application* OR software App OR video game* OR computer game* OR video recording OR videorecording* OR iPhone OR Android OR iPad OR CD-ROM OR e-learning OR elearning OR chatbot OR online chat* OR wearable device* OR wearable electronic device* OR wearable technolog* OR fitness tracker* OR activity tracker*)	436,843
S5	S3 OR S4	603,854
S6	S1 AND S2 AND S5	1,754
S7	Publication Type: Clinical Trial, Nursing Interventions, Randomized Controlled Trial	136

PsycINFO (OVID) (1806-, Search on 4 January 2025)		
#1	exp neoplasm/ or exp carcinoma/ or exp malignant neoplasm/ or (cancer* or oncolog* or neoplasm* or carcinoma* or tumo* or melanoma or sarcoma or adenocarcinoma* or glioma* or lymphoma* or myeloma* or leukemia* or leucaemia*).ti,ab.	102054
#2	exp adolescent/ or exp adolescence/ or exp young adult/ or (adolescen* or youth* or teen* or teenager* or young adult* or AYA).ti,ab.	420122
#3	exp digital health/ or exp telehealth/ or exp telemedicine/ or exp telecommunication/ or exp mobile application/ or exp internet/ or exp computers/ or exp web-based intervention/ or exp mobile phone/ or exp text messaging/ or exp video game/ or exp computer software/ or exp videoconferencing/ or exp podcast/ or exp social media/ or exp multimedia/ or exp digital video/ or exp virtual reality/ or exp wearable device/ or (digital health or telemedicine or tele-medicine or telecare or tele-care or mobile health or mhealth or m-health or telehealth or tele-health or ehealth or electronic health* or e-health or virtual or online or telehomecare or tele-homecare or telehealthcare or tele-healthcare or telemonitoring or tele-monitoring or telecommunication* or teleconference or podcast* or webcast* or email* or e-mail or videoconferenc* or video conference* or cell phone or telephone or mobile phone or smartphone or smart phone or texting* or text messag* or short messag* or SMS or computer* or internet or world wide web or website* or web browser or internet-based intervention* or internet intervention* or web-based intervention* or social media or Twitter or Facebook or Instagram or software* or computer program* or computer software or computer application or mobile application* or mobile App or portable electronic App or smartphone App or software application* or electronic application* or software App or video game* or computer game* or video recording or videorecording* or iPhone or Android or iPad or CD-ROM or e-learning or elearning or chatbot or online chat* or wearable device* or wearable electronic device* or wearable technolog* or fitness tracker* or activity tracker*).ti,ab.	466803
#4	#1 AND #2 AND #3	698
#5	(randomized controlled trial or RCT or controlled clinical trial or randomized or randomly or placebo or trial or groups or quasi experiment*).ti,ab.	825042
#6	#4 and #5	200
**ProQuest (1938-, Search on 4 January 2025)**		
S1	abstract(cancer* OR oncolog* OR neoplasm* OR carcinoma* OR tumo* OR melanoma OR sarcoma OR adenocarcinoma* OR glioma* OR lymphoma* OR myeloma* OR leukemia* OR leucaemia*) OR title(cancer* OR oncolog* OR neoplasm* OR carcinoma* OR tumo* OR melanoma OR sarcoma OR adenocarcinoma* OR glioma* OR lymphoma* OR myeloma* OR leukemia* OR leucaemia*)	3,511,636
S2	abstract(adolescen* OR youth* OR teen* OR teenager* OR young AND adult* OR aya) OR title(adolescen* OR youth* OR teen* OR teenager* OR young AND adult* OR AYA)	2,755,118
S3	abstract(digital health OR telemedicine OR tele-medicine OR telecare OR tele-care OR mobile health OR mhealth OR m-health OR telehealth OR tele-health OR ehealth OR electronic health* OR e-health OR virtual OR online OR telehomecare OR tele-homecare OR telehealthcare OR tele-healthcare OR telemonitoring OR tele-monitoring OR telecommunication* OR teleconference OR	14,486,383

	podcast* OR webcast* OR email* OR e-mail OR videoconferenc* OR video conference* OR cell phone OR telephone OR mobile phone OR smartphone OR smart phone OR texting* OR text messag* OR short messag* OR SMS OR computer* OR internet OR world wide web OR website* OR web browser OR internet-based intervention* OR internet intervention* OR web-based intervention* OR social media OR Twitter OR Facebook OR Instagram OR software* OR computer program* OR computer software OR computer application OR mobile application* OR mobile App OR portable electronic App OR smartphone App OR software application* OR electronic application* OR software App OR video game* OR computer game* OR video recording OR videorecording* OR iPhone OR Android OR iPad OR CD-ROM OR e-learning OR elearning OR chatbot OR online chat* OR wearable device* OR wearable electronic device* OR wearable technolog* OR fitness tracker* OR activity tracker*) OR title(digital health OR telemedicine OR tele-medicine OR telecare OR tele-care OR mobile health OR mhealth OR m-health OR telehealth OR tele-health OR ehealth OR electronic health* OR e-health OR virtual OR online OR telehomecare OR tele-homecare OR telehealthcare OR tele-healthcare OR telemonitoring OR tele-monitoring OR telecommunication* OR teleconference OR podcast* OR webcast* OR email* OR e-mail OR videoconferenc* OR video conference* OR cell phone OR telephone OR mobile phone OR smartphone OR smart phone OR texting* OR text messag* OR short messag* OR SMS OR computer* OR internet OR world wide web OR website* OR web browser OR internet-based intervention* OR internet intervention* OR web-based intervention* OR social media OR Twitter OR Facebook OR Instagram OR software* OR computer program* OR computer software OR computer application OR mobile application* OR mobile App OR portable electronic App OR smartphone App OR software application* OR electronic application* OR software App OR video game* OR computer game* OR video recording OR videorecording* OR iPhone OR Android OR iPad OR CD-ROM OR e-learning OR elearning OR chatbot OR online chat* OR wearable device* OR wearable electronic device* OR wearable technolog* OR fitness tracker* OR activity tracker*)	
S4	S1 AND S2 AND S3	2,506
S5	abstract(randomized controlled trial or RCT or controlled clinical trial or randomized or randomly or placebo or trial or groups or quasi experiment*) OR title(randomized controlled trial or RCT or controlled clinical trial or randomized or randomly or placebo or trial or groups or quasi experiment*)	16,183,870
S6	S4 AND S5	1,018
S7	Filters applied: Scholarly Journals	804

Supplementary Table 3. PRISMA 2020 checklist

Section and Topic	Item #	Checklist item	Location where item is reported
**TITLE**			
Title	1	Identify the report as a systematic review.	Page 1
**ABSTRACT**			
Abstract	2	See the PRISMA 2020 for Abstracts checklist.	Page 2
**INTRODUCTION**			
Rationale	3	Describe the rationale for the review in the context of existing knowledge.	Page 4
Objectives	4	Provide an explicit statement of the objective(s) or question(s) the review addresses.	Page 4
**METHODS**			
Eligibility criteria	5	Specify the inclusion and exclusion criteria for the review and how studies were grouped for the syntheses.	Page 13, Table 1
Information sources	6	Specify all databases, registers, websites, organisations, reference lists and other sources searched or consulted to identify studies. Specify the date when each source was last searched or consulted.	Page 13
Search strategy	7	Present the full search strategies for all databases, registers and websites, including any filters and limits used.	Supplementary Table 1
Selection process	8	Specify the methods used to decide whether a study met the inclusion criteria of the review, including how many reviewers screened each record and each report retrieved, whether they worked independently, and if applicable, details of automation tools used in the process.	Page 13
Data collection process	9	Specify the methods used to collect data from reports, including how many reviewers collected data from each report, whether they worked independently, any processes for obtaining or confirming data from study investigators, and if applicable, details of automation tools used in the process.	Page 14
Data items	10a	List and define all outcomes for which data were sought. Specify whether all results that were compatible with each outcome domain in each study were sought (e.g. for all measures, time points, analyses), and if not, the methods used to decide which results to collect.	Page 14, Table 2
	10b	List and define all other variables for which data were sought (e.g. participant and intervention characteristics, funding sources). Describe any assumptions made about any missing or unclear information.	Page 14, Table 2
Study risk of bias assessment	11	Specify the methods used to assess risk of bias in the included studies, including details of the tool(s) used, how many reviewers assessed each study and whether they worked independently, and if applicable, details of automation tools used in the process.	Page 14

Section and Topic	Item #	Checklist item	Location where item is reported
Effect measures	12	Specify for each outcome the effect measure(s) (e.g. risk ratio, mean difference) used in the synthesis or presentation of results.	Page 14
Synthesis methods	13a	Describe the processes used to decide which studies were eligible for each synthesis (e.g. tabulating the study intervention characteristics and comparing against the planned groups for each synthesis (item #5)).	Page 14–15
	13b	Describe any methods required to prepare the data for presentation or synthesis, such as handling of missing summary statistics, or data conversions.	Page 14
	13c	Describe any methods used to tabulate or visually display results of individual studies and syntheses.	Page 14–15
	13d	Describe any methods used to synthesize results and provide a rationale for the choice(s). If meta-analysis was performed, describe the model(s), method(s) to identify the presence and extent of statistical heterogeneity, and software package(s) used.	Page 14
	13e	Describe any methods used to explore possible causes of heterogeneity among study results (e.g. subgroup analysis, meta-regression).	Page 14
	13f	Describe any sensitivity analyses conducted to assess robustness of the synthesized results.	Page 14
Reporting bias assessment	14	Describe any methods used to assess risk of bias due to missing results in a synthesis (arising from reporting biases).	Page 14
Certainty assessment	15	Describe any methods used to assess certainty (or confidence) in the body of evidence for an outcome.	Not Applicable – no evaluation of evidence conducted
**RESULTS**			
Study selection	16a	Describe the results of the search and selection process, from the number of records identified in the search to the number of studies included in the review, ideally using a flow diagram.	Page 4
	16b	Cite studies that might appear to meet the inclusion criteria, but which were excluded, and explain why they were excluded.	Page 4, Figure 1
Study characteristics	17	Cite each included study and present its characteristics.	Page 4–5
Risk of bias in studies	18	Present assessments of risk of bias for each included study.	Page 5–6
Results of individual studies	19	For all outcomes, present, for each study: (a) summary statistics for each group (where appropriate) and (b) an effect estimate and its precision (e.g. confidence/credible interval), ideally using structured tables or plots.	Page 6–7, Figure 4
Results of	20a	For each synthesis, briefly summarise the characteristics and risk of bias among contributing studies.	Page 5–6, Figure 3

Section and Topic	Item #	Checklist item	Location where item is reported
syntheses	20b	Present results of all statistical syntheses conducted. If meta-analysis was done, present for each the summary estimate and its precision (e.g. confidence/credible interval) and measures of statistical heterogeneity. If comparing groups, describe the direction of the effect.	Page 6–7, Figure 4
	20c	Present results of all investigations of possible causes of heterogeneity among study results.	Page 6–7, Supplementary Table 2
	20d	Present results of all sensitivity analyses conducted to assess the robustness of the synthesized results.	Supplementary Figure 1–7
Reporting biases	21	Present assessments of risk of bias due to missing results (arising from reporting biases) for each synthesis assessed.	Page 5–6
Certainty of evidence	22	Present assessments of certainty (or confidence) in the body of evidence for each outcome assessed.	Not Applicable – no evaluation of evidence conducted
**DISCUSSION**			
Discussion	23a	Provide a general interpretation of the results in the context of other evidence.	Page 9
	23b	Discuss any limitations of the evidence included in the review.	Page 12
	23c	Discuss any limitations of the review processes used.	Page 12
	23d	Discuss implications of the results for practice, policy, and future research.	Page 12–13
**OTHER INFORMATION**			
Registration and protocol	24a	Provide registration information for the review, including register name and registration number, or state that the review was not registered.	Page 13
	24b	Indicate where the review protocol can be accessed, or state that a protocol was not prepared.	Page 13
	24c	Describe and explain any amendments to information provided at registration or in the protocol.	Not applicable
Support	25	Describe sources of financial or non-financial support for the review, and the role of the funders or sponsors in the review.	Page 15
Competing interests	26	Declare any competing interests of review authors.	Page 15
Availability of data, code and other materials	27	Report which of the following are publicly available and where they can be found: template data collection forms; data extracted from included studies; data used for all analyses; analytic code; any other materials used in the review.	Page 15

From: Page MJ, McKenzie JE, Bossuyt PM, Boutron I, Hoffmann TC, Mulrow CD, et al. The PRISMA 2020 statement: an updated guideline for reporting systematic reviews. BMJ 2021;372:n71. doi: 10.1136/bmj.n71. This work is licensed under CC BY 4.0. To view a copy of this license, visit https://creativecommons.org/licenses/by/4.0/
